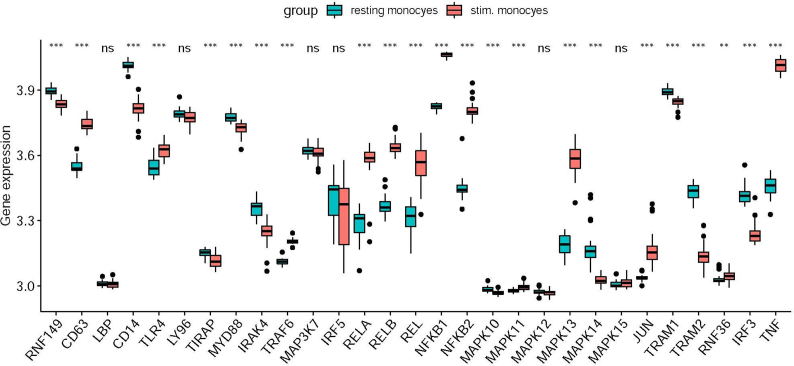# RNF149 negatively regulates LPS/TLR4 signal transduction by ubiquitination-mediated CD63 degradation

**DOI:** 10.1016/j.heliyon.2024.e34350

**Published:** 2024-07-11

**Authors:** Xiu-An Yang, Yingying Wang, Mingyu Gong, Zicheng Zhao, Fengchun Lv, Xiaoyu Zhang, Yan Li

**Affiliations:** a Laboratory of Genetic Engineering and Genomics, School of Basic Medical Sciences, Chengde Medical University, Chengde 067000, China; b Hebei Key Laboratory of Nerve Injury and Repair, Chengde Medical University, Chengde 067000, China; c Department of Biomedical Engineering, Chengde Medical University, Chengde 067000, China; d Graduate School of Chengde Medical University, 067000 Chengde, China; e Institute of Biophysics, Chinese Academy of Sciences, Beijing 100101, China

**Keywords:** RNF149, Ubiquitin ligase, CD63, Tetraspanins, LPS/TLR4 signal transduction

## Abstract

This study aims to investigate the role of RNF149 and tetraspanin CD63 in lipopolysaccharide/Toll-like receptor 4 (LPS/TLR4) signal transduction. TNF-α was assessed using enzyme-linked immunosorbent assay. The distribution of TLR4 was examined through flow cytometry after CD63 knockdown. Real-time polymerase chain reaction was used to analyze the expression of the target genes RNF149 and CD63 under different conditions. Western blotting was employed to detect gene expression, while immunoprecipitation and confocal microscopy were used to evaluate protein interactions. Transcriptome array data from stimulated monocytes (GSE7547) was obtained from GEO and subjected to bioinformatic analysis. It is suggested that CD63 may serve as a substrate of RNF149, with RNF149 capable of directly interacting with CD63. RNF149 degrades CD63 through covalent modification of CD63 at lysine 29 of the ubiquitin monomer, leading to the formation of a multiubiquitin chain. Both RNF149 and CD63 interact with TLR4, with CD63 promoting LPS/TLR4 signaling and RNF149 inhibits it. CD63 does not impact the distribution of TLR4 on the cell surface and does not directly interact with TIRAP, IRAK4, or TRAF6, but does interact with Myd88.RNF149 plays a negative regulatory role in LPS/TLR4 signal transduction by mediating ubiquitination-induced CD63 degradation.

## Graphical abstract

Significant differences in the transcription levels of most genes involved in the LPS/TLR4 signal pathway after stimulation, including TLR4, MYD88, TIRAP, TRAM1, TRAM2, JUN (AP-1), and NF-κB factors. RNF149 decreased while CD63 increased in monocytes after stimulation with LPS. RNF149 and CD63 are highly involved in LPS/TLR4 signal transduction.

**Figure:**
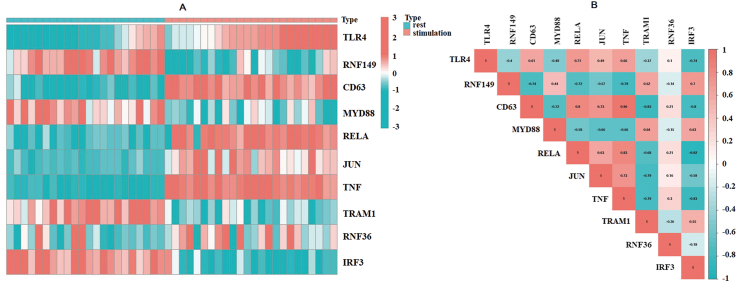

## 1 Introduction

Ubiquitylation, a three-enzyme cascade consisting of the conjugation of a highly conserved 76-amino-acid protein ubiquitin (Ub) to lysine residues, is one of the most common posttranslational modifications [1]. Ub-activating enzymes (E1s), Ub-conjugating enzymes (E2s), and Ub ligases (E3s) are essential in the process of ubiquitylation [2]. The efficiency and substrate specificity of the ubiquitination cascade depends on E3s, which transfers Ub from E2 to a primary amine for target proteins [3]. E3s are classified into three types: the really interesting new gene RING finger proteins, the homologous to E6-AP C-terminus (HECT) proteins, and the RING-in-between-RING (RBR) proteins [4].

CD4 T-cell unresponsiveness to peripheral tolerance is maintained by an important mechanism called anergy [5]. The E3s of Cbl-b, Itch, and GRAIL have been documented to be involved in T-cell anergy [6]. GRAIL (gene related to anergy in lymphocytes, also known as RNF128) was first reported as an E3 ubiquitin ligase that inhibits cytokine gene transcription in anergic CD4^+^ T cells by Anandasabapathy et al. [7]. GRAIL belongs to the PA-TM-RING family, which consists of a protease-associated (PA) domain and a RING finger domain separated by a transmembrane (TM) domain [8]. In humans, 11 proteins are known to belong to the PA-TM-RING family [9]. To date, RNF128 [10] and RNF13 [11] are the most extensively studied members of the PA-TM-RING family.

RNF128 is upregulated upon induction of T-cell anergy, while RNF13 is documented to suppress myoblast proliferation and is upregulated in neurons, indicating diverse physiological roles of the PA-TM-RING family [12]. Hong et al. previously reported that RNF149 is an E3 ubiquitin ligase that acts on wild-type v-Raf murine sarcoma viral oncogene homolog B1 (BRAF) [13]. We demonstrated that RNF149 is located in lysosomes and regulates cell proliferation by polyubiquitination mediated CD9 degradation [14]. In this study, the characteristics of RNF149 as an E3 ligase were further studied. We found that RNF149 negatively regulates lipopolysaccharide/Toll-like receptor 4 (LPS/TLR4) signal transduction by ubiquitination-mediated CD63 degradation.

## 2 Materials and methods

### 2.1 Reagents

The Trizol Reagent and Lipofectamine® 2000 Transfection Reagent were purchased from Invitrogen (Carlsbad, CA, USA). The SYBR Green PCR Master Mix was provided by Applied Biosystems (San Francisco, CA). The protease inhibitors (PMSF, Aprotinin, Leupeptin, Pepstain) were acquired from AMRESCO (Solon, OH, USA) and cocktail was provide by AbMole. The protein A/G beads were purchased from Santa Cruz Biotechnology (Santa Cruz, CA, USA). LysoTracker Red, ER-Tracker Red and the other organelle probes were purchased from Molecular Probes (Eugene, OR, USA). Puromycin, the proteasome inhibitor MG132, transfection reagent PEI, goat anti-mouse IgG-HRP, goat anti-rabbit IgG-HRP, anti-flag, and other antibodies were obtained from Sigma–Aldrich (St. Louis, MO, USA). The sheep anti-mouse IgG Fc segment-HRP was purchased from Thermo Fisher Scientific (Waltham, MA, USA). The Anti-HA antibody was provided by Beijing Zhongshan Jinqiao Biotechnology Co., Ltd (Beijing, China). The anti-CD63 primary antibody was acquired from Abcam (Cambridge, MA, USA). The ECL substrate color developing solution was purchased from GE Healthcare (Little Chalfont, Buckinghamshire, UK). The Moloney Murine Leukemia Virus Reverse Transcriptase (M-MLV) was obtained from Promega (Madison, WI, USA).

### 2.2 Plasmids

The PCI-neo mammalian expression vector used in this study was purchased from Promega (Madison, WI, USA). The coding sequences (CDSs) of the genes used in this study were cloned from the complementary DNA (cDNA) library of the 293T cell line. Plasmids pRFP-C1-CD63, PCI-RNF149-eGFP-C, PCI-CD63-eGFP-C, PCI-RNF149-HA-C, PCI-CD63, PCI-Ub-Myc-N, PCI-UbK48R-Myc-N, and PCI-UbK63R-Myc-N were constructed by inserting amplified human-derived gene fragments into PCI-neo vectors. The stop codon of the inserted gene CDS region was removed to express different tags with the plasmids. For example, in the plasmid PCI-UbK48R-Myc-N, the 48th lysine of the human Ub gene was mutated into arginine and fused with an N-terminal Myc tag. The shRNA and control sequences were inserted into the pSUPER.retro.puro vector (OligoEngine, Seattle, Washington, USA) to knock out RNF149.

### 2.3 Cell culture and transfection

The human embryonic kidney Line 293T (293T), human cervical cancer cell line HeLa, and mouse Raw264.7 cells were used in this study. The cells were cultured in high-glucose Dulbecco's modified Eagle's medium (DMEM, Gibco; Thermo Fisher Scientific, Inc.) supplemented with 10 % fetal bovine serum (FBS, Gibco, Thermo Fisher Scientific, Inc.) at 5 % CO_2_ and 37 °C. To maintain the stable characteristics of 293T cells, 2.5 mg/L puromycin was added to the culture medium. Raw264.7 cells were cultured in endotoxin-free DMEM.

The Lipofectamine® 2000 Transfection Reagent was used for plasmid transfection, and the siRNA Transfection Reagent was used for siRNA transfection. Transient transfection was performed following the manufacturer's instructions. Briefly, HeLa cells intended for immunofluorescence colocalization were seeded at a density of 3 × 10^5^ cells in a 12-well plate and allowed to grow for 12–18 h prior to transfection. Subsequently, the cells were transfected with 1 μg of each plasmid and 4 μl of the transfection reagent. After an incubation period of 4–6 h, the transfection solution was replaced with fresh cell culture medium, and functional tests were performed following an additional incubation of 24–36 h.

### 2.4 Enzyme-linked immunosorbent assay (ELISA)

The TNF-α (eBioscience, #29-8329-65) kit were acquired from Thermo Fisher Scientific (Waltham, MA, USA), and the _TNF-α_ content in the cell culture supernatant was detected following the manufacturer's instructions. Briefly, the coated antibody was diluted with the coating solution according to the recommended dilution ratio. Subsequently, 100 μL of the prepared solution was added to each well of a 96-well plate and incubated at 4 °C overnight. The plate was washed 4 times with 1 × PBSTand then blocked with ELISA diluent solution for 1 h at room temperature. After two washes with 1 × PBST, the cell culture supernatant (100 μL per well) was diluted and incubated at 25 °C for 2 h. The wells were thoroughly washed four times with 1 × PBST and incubated with the biotinylated antibody for 1 h. Following another wash, the plate was incubated with the HRP-labeled secondary antibody (100 μl/well) for 1 h at room temperature. Finally, the plate was developed with 100 μl of the eBioscience TMB solution, and the absorbance at 450 nm was measured.

### 2.5 Flow cytometry

After knocking down CD63 in cells using siRNA, the transfected RAW264.7 cells were collected, washed and incubated with 1 μg of anti-mouse CD63 at room temperature for 10 min. Then the cells were incubated with 0.5 μg of anti-mouse TLR4 APC at room temperature for 15 min followed by washing with PBS three times. After removing the supernatant, the cells resuspended in 300 μl of PBS solution, and detected by flow cytometry.

### 2.6 Real-time polymerase chain reaction (RT–PCR)

Total RNA was extracted from RAW264.7 cells with different statuses using the TRIzol reagent strictly following the manufacturer's instructions. Subsequently, cDNA was synthesized from 1 μg of total mRNA using reverse transcriptase M-MLV. The SYBR Green PCR Master Mix was utilized to quantify the expression of mRNAs. The expression of the target genes RNF149 and CD63 was normalized to the relative expression of GAPDH. The RT-PCR primers used were as follows: RNF149-mouse-SYB forward, 5′-TCAGCGGTCAGTCTGTGGT-3′ and RNF149-mouse-SYB reverse, 5′-GGCCAATAACCTTCTTAGTCTCC′; CD63-mouse-SYB forward, 5′-TCAACATAACTGTGGGCTGTGGGA and CD63-mouse-SYB reverse, 5′-AGCCACCAGCAGTATGTTCTTCCT-3′; GADPH-mouse-SYB forward, 5′-ATCAACGACCCCTTCATTGACC-3′ and GADPH-mouse-SYB reverse, 5′-CCAGTAGACTCCACGACATACTCAGC-3′. RT–PCR was performed using an Applied Biosystem 7300 Real-Time PCR System (Applied Biosystems; Thermo Fisher Scientific, Inc.). The resulting PCR underwent a melting curve analysis, and the relative gene expression data of the target genes were analyzed by using the 2^−ΔΔCt^ method.

### 2.7 Western blot (WB)

After a certain period of cultivation, the cells were collected and lysed with the RIPA buffer. Cell lysis was then loaded onto sodium dodecyl sulfate-polyacrylamide gel electrophoresis (SDS-PAGE) on 12 % acrylamide minigels (Bio-Rad) and transferred to a PVDF membrane. The membrane was subsequently blocked with 5 % skim milk at room temperature for 1 h. After washing with 1 × TBST, the membrane was probed with the primary antibodies at 4 °C overnight. The recommended concentrations of horseradish peroxidase-conjugated secondary antibodies were then added, and the membranes were incubated for 40 min at room temperature. Finally, immunoreactivities were visualized by using enhanced chemiluminescence according to the manufacturer's instructions (Thermo Scientific, Waltham, Massachusetts, USA).

### 2.8 Immunoprecipitation (IP)

After 36 h of cell transfection, the 293T cells were harvested and lysed with 600 μl of the pre-cooled IP buffer containing PMSF on ice for 30 min. The cell lysate was then centrifuged at 12,000 rpm at 4 °C for 15 min, and the supernatant was collected. Then, 1 μg of the antibody and 15 μl of the protein A/G beads were added to 40 μl of the supernatant in sequence and incubated at 4 °C for 2 h. After washing with the precooled IP buffer 5 times, the samples were collected at 3,000 rpm at 4 °C for 5 min, separated by SDS-PAGE, and analyzed by WB with the corresponding antibodies.

### 2.9 *In vivo* ubiquitination assays

MG132 was added 4 h before cell lysis until the final concentration reached 10 μm/ml. After washing twice with the precooled PBS, the cells were lysed with 270 μl of the IP buffer B (50 mmol/L Tris-HCl, pH 8.0, 150 mmol/L NaCl, 1 mmol/L EDTA, 1 × protease inhibitor cocktail) and 30 μl of the 5 % SDS. After boiling for 15 min, total cell lysates were centrifuged at 13,000 rpm for 10 min, and 5 % of the supernatant was taken for WB detection. The remaining supernatants were diluted to 10× with IP buffer A (50 mmol/L Tris-HCl pH 7.4, 150 mmol/L NaCl, 1 mmol/L EDTA, 0.5 % NP40, 5 mmol/L MgCl2). The sample was incubated with 1 μg of the anti-Flag antibody at 4 °C for 2 h, followed by an additional 2 h incubation with 15 μl of the protein A-agarose beads. The beads were collected by centrifugation at 3000 rpm at 4 °C. Finally, 50 μl of the protein samples were boiled and ubiquitin adducts were detected by WB using an anti-Ub antibody.

### 2.10 Bioinformatics analysis

The transcriptome array data of stimulated monocytes (GSE7547) was downloaded from GEO (https://www.ncbi.nlm.nih.gov/gds/?term=).

Bioinformatics analysis was performed using the R programming language (https://www.r-project.org/). Samples of resting monocytes and stimulated monocytes of good angiogenic responders were selected from the gene set and divided into two groups. The boxplot of characteristic genes was created using the ggboxplot function in the “ggplot 2” package, and the differences in gene expression among the different groups were compared using the wilcox.test. The “heatmap” package was utilized to standardize the characteristic genes, and a “complete” clustering method was employed to generate the heatmap. The Pearson correlation between pairwise characteristic genes was calculated to obtain the correlation coefficient, and the correlation heatmap was produced using the corrplot function in the “corrplot” package. The network diagram of characteristic genes was created using the network_plot function of the “corrr” package.

## 3 Results

### 3.1 The residue E7 of the signal peptide and TM are important for the location of RNF149

**Fig. 1:**
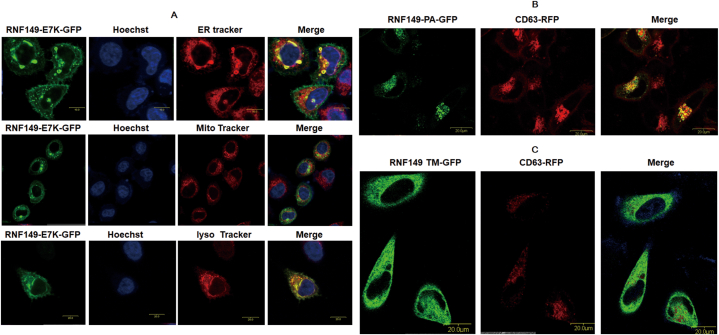
Identification of key factors related to RNF149 positioning. (A) E7K mutation on the peptide disrupted the specific location of RNF149. (B) PA domain deletion had no influence on the positioning of RNF149. (C) TM deletion disrupted the specific location of RNF149.

Through the overexpression of the exogenous RNF149 plasmid and staining of endogenous organelles, we previously showed that RNF149 is a transmembrane protein located on lysosomes [14]. Somatic mutation of RNF149 (c.19G > A, p.E7K) was identified in breast cancers by Sjöblom et al. [15]. To further investigate the importance of this site in RNF149 location, an E7K mutation was constructed, and the location of RNF149 was investigated. Our results indicated that RNF149 no longer localized to lysosomes but dispersed throughout the entire cell. This suggests that the residue E7 of the signal peptide is important for the specific location of RNF149 (Fig. 1A). To assess the effects of different domains on the localization of RNF149 cells, the interaction between RNF149 truncations (PA domain deletion or TM deletion) and CD63 was investigated. As shown in Fig. 1BCE, following the deletion of PA domain, RNF149 could still be well localized on lysosomes. However, after the deletion of TM domain, the localization was no longer specific and dispersed throughout the entire cell. The results indicate that the TM domain, rather than the PA domain, plays a significant role in the positioning of RNF149.

### 3.2 RNF149 co-localizes and interacts with CD63

**Fig. 2:**
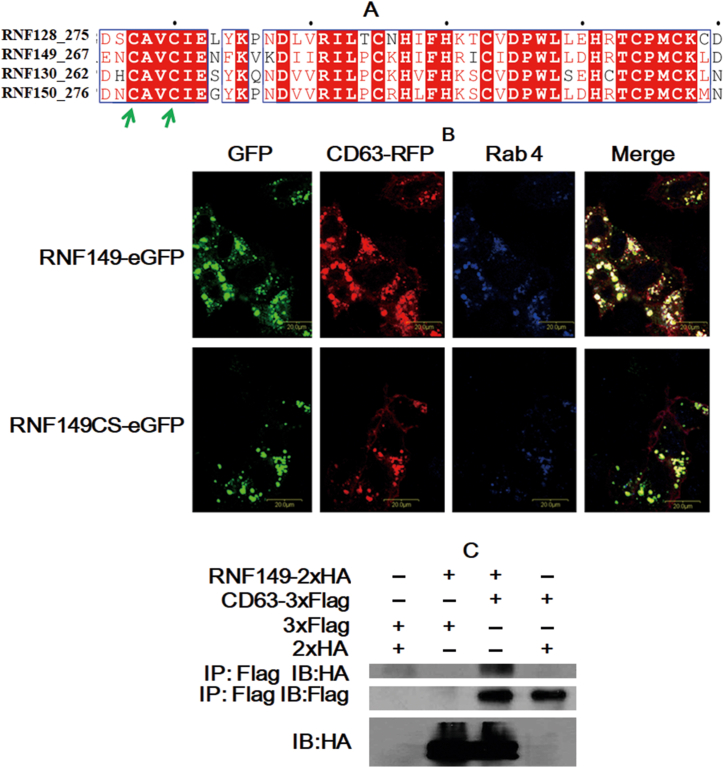
RNF149 colocalizes and interacts with CD63. (A) Sequence alignment of RNF149CS mutation sites (green arrow) and its vicinity. (B) HeLa cells were transiently cotransfected with PCI-RNF149-eGFP and PCI-CD63-RFP plasmids and labeled with an antibody against the endocytosis labeling protein Rab4. The PCI-RNF149-eGFP or PCI-CD63-RFP plasmid was transiently transfected into HeLa cells by Lipofectamine 2000. The localization of GFP and RFP was observed by laser confocal fluorescence microscopy, and the results showed that they were located next to each other. (C) 293T cells were transiently cotransfected with different plasmid combinations and lysed 24 h after transfection. The binding of RNF149 to CD63 was detected by immunoprecipitation using an anti-Flag antibody and an anti-HA antibody. (For interpretation of the references to color in this figure legend, the reader is referred to the Web version of this article.)

Previous studies have shown that CD151 [16] and CD81 [17], members of the tetraspanin protein family, are substrates of RNF128 ubiquitination. RNF149 and RNF128 belong to the same family and have similar structures. Therefore, we hypothesized that the substrate of RNF149 may also be a member of the tetraspanin family. Preliminary screening of 22 tetraspanin family molecules by flow cytometry showed that CD63 may be a substrate of RNF149 (data not shown). To verify whether RNF149 and CD63 are colocalized in cells, PCI-RNF149-eGFP-C, PCI-RNF149CS-eGFP-C (C269S/C272S, Fig. 2A), and PCI-CD63-RFP-C plasmids were transfected into HeLa cells and observed by fluorescence confocal microscopy. Twenty-four hours after transfection, endosomes were labeled with Rab4. As shown in Fig. 2B, the distribution of RNF149-eGFP only had negligible differences from that of CD63-RFP. After labeling the endocytosis with the Rab4 antibody, it was found that RNF149 and CD63 were colocalized. The colocalization of the RNF149CS mutant and CD63 did not change, nor did the distribution of the RNF149CS-eGF fusion protein. This indicated that the RING domain mutation of RNF149 had little effect on the colocalization with CD63. To further verify the direct interaction between RNF149 and CD63, plasmids of PCI-CD63-3 × Flag-C, PCI-RNF149-2 × HA-C, and control tags were cotransferred into H293T cells and detected by IP after 24 h of culture. The positive group could identify the corresponding bands, while the control group did not find the bands (Fig. 2C, Supplementary Fig. S2C1-2). Together, our results showed that RNF149 could interact with CD63. The PA domain of the single subunit transmembrane RNF128 captures the large extracellular loop domain of tetraspanin family molecules of CD151 and CD81 across the cell membrane [16,17]. Accordingly, the interaction between RNF149 and CD63 might also be accomplished by the combination of the PA region of RNF149 and the EC2 region of CD63.

### 3.3 RNF149 ubiquitinates CD63 through lysine at amino acid 29 of the Ub monomer and leads to CD63 proteolysis

**Fig. 3:**
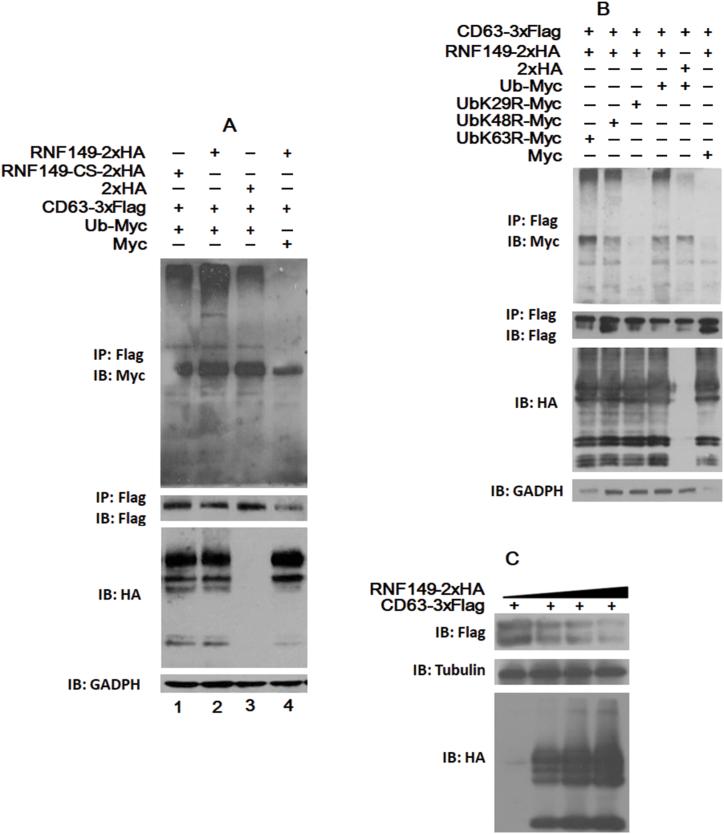
RNF149 ubiquitinates CD63 through lysine at amino acid 29 of the Ub monomer and leads to CD63 proteolysis. (A) 293T cells were cotransfected with Ub-myc, CD63-3 × Flag, and different combinations of RNF149-2 × HA, RNF149-CS-2 × HA, and control plasmids. 293T cells were cotransfected with RNF149-2 × HA, CD63-3 × Flag, and different combinations of Ub-Myc, UbK48R-Myc, UbK63R-Myc, UbK29R- Myc, and control plasmids. (B) The CD63-3 × Flag plasmids and different concentrations of the RNF149-2 × HA plasmids were transfected into HeLa cells. (C)After the cells were lysed, immunoprecipitation was performed using the corresponding antibodies.

RING-structured E3 lipases can ubiquitinate the substrate or synthesize multiubiquitinated chains on the substrate through different lysine residues on ubiquitin, thus playing related functions [18]. We previously reported that RNF149 regulates cell proliferation by polyubiquitination-mediated CD9 degradation [14]. In this study, we discovered that RNF149 colocalizes and interacts with CD63. Based on this observation, we hypothesize that RNF149 may also induce CD63 degradation. To investigate whether CD63 is a substrate of RNF149 and determine the substrate ubiquitination pattern, we conducted ubiquitination experiments. H293T cells were transfected with various combinations of Ub-myc, PCI-CD63-3 × Flag-C, PCI-RNF149-2 × HA-C, RNF149-CS-2 × HA, and control plasmids to study the effect of the RING domain of RNF149 on CD63 ubiquitination. Thirty-six hours after cell transfection and 4 h before cell lysis, MG132 at a final concentration of 10 μmol/ml was added, and IP was performed. Compared with the wild-type RNF149, multiubiquitination band of CD63 was weakened in the RNF149 RING domain mutant (RNF149-CS) group (Fig. 3A, Supplementary Figs. 3A1–4).). This finding indicates that the RING domain of RNF149 plays a crucial role in the ubiquitination of CD63. When lysine at position 29 of Ub was mutated to arginine, the multiubiquitination band of CD63 disappeared (Fig. 3B, Supplementary Fig. S3B1–4).). The results indicate that the ubiquitination of CD63 by RNF149 occurs through covalent modification of CD63 by lysine at position 29 of the ubiquitin monomer and the formation of a multiubiquitin chain. According to the theory proposed by Pickart et al., the ultimate outcome of the ubiquitination of CD63 by RNF149 may lead to proteolysis [18]. PCI-CD63-3 × Flag-C and various concentrations of PCI-RNF149-2 × HA-C were transferred into HeLa cells, and the levels of CD63 were assessed by IP. As shown in Fig. 3C (Supplementary Fig. S3C-1,2).**)**, as the expression of RNF149 increased, the expression of CD63 decreased gradually, suggesting that RNF149 can degrade CD63 through ubiquitination.

### 3.4 RNF149 and CD63 are associated with LPS/TLR4 signal transduction

**Fig. 4:**
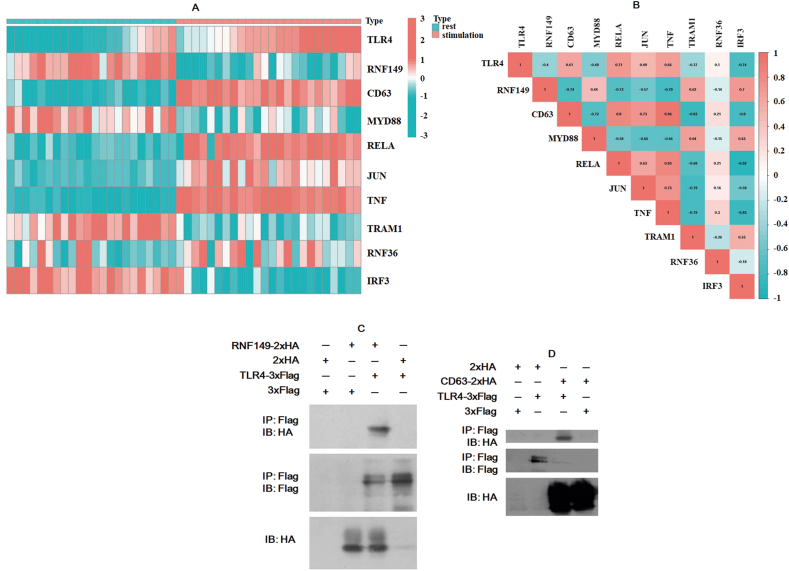
RNF149 and CD63 are associated with LPS/TLR4 signal transduction. (A) Heatmap of the expression signature of LPS/TLR4 signal pathway representative genes from transcriptome array data (GSE7547). (B) Correlation results of LPS/TLR4 signal transduction key genes. TLR4-3 × Flag was cotransfected with RNF149-2 × HA (C) or CD63-2 × HA (D) and the corresponding control plasmids. After the cells were lysed, immunoprecipitation was performed using the corresponding antibodies.

Through the aforementioned investigations, we have confirmed that CD63 is the substrate of RNF149. However, it remains unclear in which physiological process RNF149 ubiquitinates CD63. Previously, we demonstrated that RNF149 can bind to the tetraspanin CD9, and in this study, we have also found that RNF149 can bind to CD63 [14]. Both CD9 and CD63 are exosome markers and have been reported to significantly increase in macrophages when stimulated with lipopolysaccharide (LPS) [19]. Based on these findings, we suspect that RNF149 may be associated with LPS/TLR4 signal transduction [20]. To test this hypothesis, we downloaded transcriptome array data of stimulated monocytes (GSE7547) from GEO and conducted bioinformatics analysis. Detailed information about the samples can be found in the study by Schirmer et al. [21]. We observed significant differences in the transcription levels of most genes involved in the LPS/TLR4 signal pathway after stimulation, including TLR4, MYD88, TIRAP, TRAM1, TRAM2, JUN (AP-1), and NF-κB factors (Supplementary Fig. 1). Notably, RNF149 decreased while CD63 increased in monocytes after stimulation with LPS. The heatmap in Fig. 4A illustrates the expression signature of representative genes from the LPS/TLR4 signal pathway in each sample before and after LPS stimulation. Correlation analysis revealed a high association (r ≥ 0.4) between TLR4 and RNF149, CD63, MYD88, RELA, and JUN (AP-1) as shown in Fig. 4B. Therefore, our results indicate that RNF149 and CD63 are highly involved in LPS/TLR4 signal transduction.

As RNF149, CD63, and TLR4 are all membrane proteins, we speculate that there may be interaction between them. To test the interaction between TLR4 and RNF149, PCI-TLR4-3_×_Flag-C, PCI-RNF149-2_×HA-C_, and the control plasmids were cotransfected into 293T cells. In the results, the specific band of RNF149 could be detected by IP in the experimental group but not in the control group (Fig. 4C, Supplementary Fig. S4C1–3).), indicating that RNF149 interacts with TLR4. Similarly, PCI-TLR4-3_×_Flag-C, PCI-CD63-2 × HA-C, and the control plasmids were cotransfected into 293T cells and detected by IP to reveal the interaction between CD63 and TLR4. As shown in Fig. 4D (Supplementary Fig. S4D1–2), there is indeed an interaction between CD63 and TLR4. We previously demonstrated that RNF149 could bind to the tetraspanin CD9 [14]; however, we found that CD9 could not bind to TLR4 (data not shown).

### 3.5 CD63 or RNF149 knockdown in RAW264.7 cells affects the production of TNF-α stimulated by LPS

**Fig. 5:**
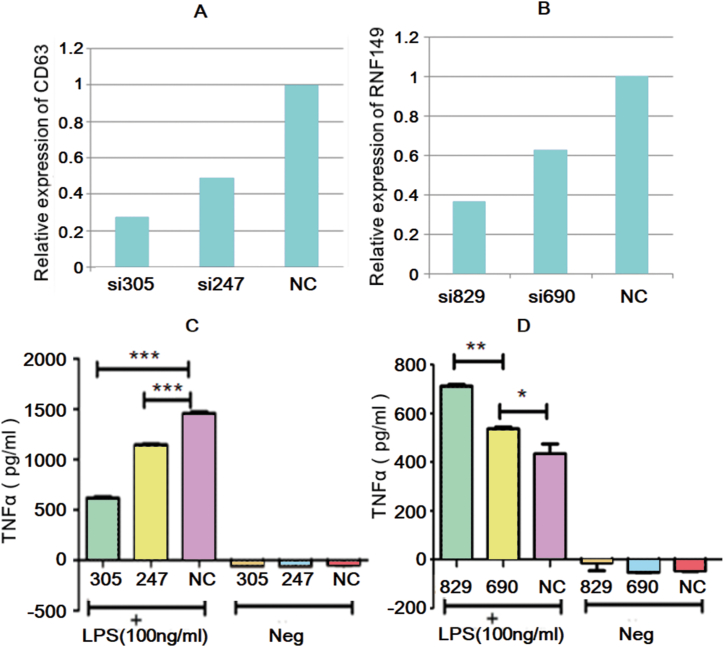
CD63 or RNF149 knockdown in RAW264.7 cells affects the production of TNF-α stimulated by LPS. When RNF149 (A) and CD63 (B) were knocked out in RAW264.7 cells and stimulated by LPS, the level of TNF-α in the CD63 knockdown group was lower (C), while that in the RNF149 knockdown group (D) was higher than that in the negative control group.

To further investigate the relationship between RNF149, CD63, and the TLR4 signaling pathway, a siRNA test was conducted in mouse RAW264.7 cells. The cells were transfected with RNF149 or CD63 siRNA (TurboFect siRNA, Fermentas) and incubated for 24 h. After replacing the culture medium with a fresh one, LPS was added to the culture medium of the experimental group to a final concentration of 100 ng/ml, and the cells were cultured for an additional 2 h. Subsequently, the mRNA expression of RNF149 and CD63 was analyzed by RT-PCR, and the content of TNF-α was detected by ELISA. The expression of RNF149 and CD63 decreased in the experimental group, indicating successful knockdown (Fig. 5AB). Following stimulation of RAW264.7 cells with LPS, the TNF-α level in the CD63 knockdown group was lower than that in the negative control group (Fig. 5C). Conversely, the TNF-α level detected after RNF149 knockdown was higher than that in the negative control group (Fig. 5D).

### 3.6 CD63 interacts with the TLR4 downstream linker protein Myd88

**Fig. 6:**
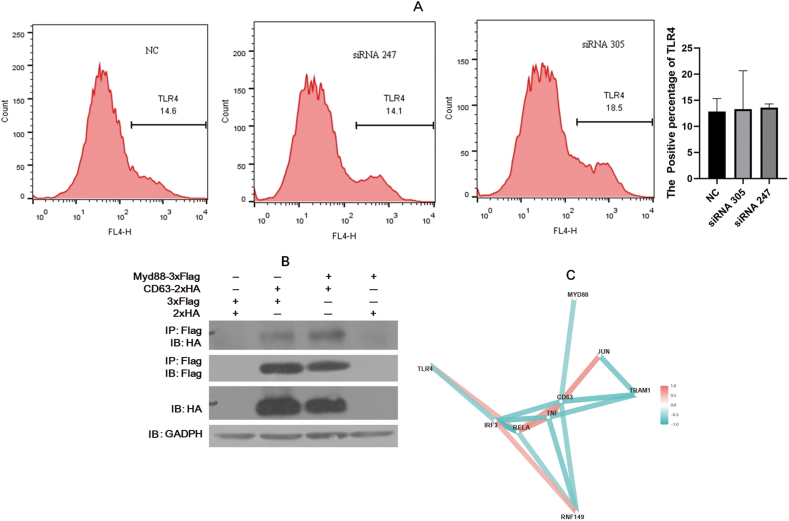
CD63 interacts with the TLR4 downstream linker protein Myd88. (A) Flow cytometry of the cell ratio and Geomange value of the TLR4-positive cells in CD63 siRNA know-out RAW264.7 cells 24 h after transfection. (B) The PCI-CD63-2 × HA-C plasmid was cotransfected with pCI-Myd88-3 × Flag-C, in 293T cells and detected by immunoprecipitation. (C) Network diagram of related data frames was constructed using the expression level of LPS/TLR4 signaling pathway genes. The correlation value was larger than 0.7. Red line indicates positive association while cyan line indicates negative association. (For interpretation of the references to color in this figure legend, the reader is referred to the Web version of this article.)

The aforementioned results suggest that CD63 plays a role as a promoting factor in LPS/TLR4 signaling, while RNF149 functions as an inhibiting factor. According to previous studies, there may be two potential mechanisms. First, as a transporter-related physiological activity, CD63 may influence the distribution of TLR4 on the cell surface through the endocytosis of TLR4 [22]. To test this hypothesis, CD63 was knocked out by siRNA in RAW264.7 cells. Twenty-four hours after transfection, TLR4 was labeled with the developed TLR4-APC antibody on the cell surface and detected with the TLR4 flowing antibody (Fig. 6A). When CD63 was knocked out, the cell ratio and Geomange value of the TLR4-positive cells negligibly changed, indicating that CD63 does not affect the distribution of TLR4 on the cell surface. Another possible mechanism might be that CD63 promotes signal transduction of TLR4 by recruiting protein molecules downstream of TLR4 through certain intracellular proteins [23], while RNF149 indirectly regulates the TLR4 signal pathway through the multiubiquitination degradation of CD63. TLR4 helps maintain the balance of immune cell viability and inflammation through the MyD88-dependent pathway and the MyD88-independent pathway [24]. In the MyD88-dependent pathway, IL-1 receptor-associated kinase (IRAK) is recruited to TLR4 by MyD88, ultimately activating JUN (AP-1) and NF-κB [25]. The MyD88-independent pathway is initiated by TRAM and TRIF (RNF36), stimulating the transcription factor interferon regulatory Factor 3 (IRF-3) [26]. Endocytosis of TLR4 is crucial for the MyD88-independent pathway [27], and no change in TLR4 distribution were observed. Therefore, we hypothesize that CD63 may regulate the TLR4 signaling pathway through the MyD88-dependent pathway. To prove this hypothesis, we transfected the PCI-CD63-2 × HA-C plasmid and TLR4 downstream gene expression plasmids pCI-TIRAP-3 × Flag-C, pCI-Myd88-3 × Flag-C, pCI-IRAK4-3 × Flag-C, and pCI-TRAF6-3 × Flag-C into 293T cells. IP after 24 h reveled that CD63 did not directly interact with TIRAP, IRAK4 or TRAF6 (data not shown) but interacted with Myd88 (Fig. 6B, Supplementary Fig. S6B1-2). Therefore, CD63 may bind to the linker protein Myd88 in the LPS/TLR4 signaling pathway through the C-terminal tail domain of the intracellular region and play a role similar to that of the TIRAP molecule in the LPS/TLR4 signaling process, thereby promoting the cytokine production. A network diagram of related data frames was constructed using the expression levels of LPS/TLR4 signaling pathway genes (r ≥ 0.7, Fig. 6C). The expression of TLR4, JUN (AP-1), and NF-κB exhibited a positive association with the expression of TNF. It is noteworthy that the expression of Myd88 was negatively associated with the expression of CD63.

## 4 Discussion

RNF13, a well-studied PA-TM-RING family molecule, has been found to have diverse functions, including roles in immune responses [11], the nervous system [28], and endoplasmic reticulum stress [29]. Given this, it is suggested that the PA-TM-RING family of molecules may have multiple functions. Both our study and that of Hong et al. have demonstrated that RNF149, as an E3 ubiquitin ligase, targets CD9 [14] and BRAF [13]. In this study, we have uncovered an interaction between RNF149 and CD63. Furthermore, both RNF149 and CD63 have the ability to bind to TLR4. Moreover, the binding of CD63 to Myd88 suggests that RNF149 regulates LPS/TLR4 signal transduction by promoting the degradation of CD63 through ubiquitination.

Immunofluorescence analysis revealed that RNF13 is located in the endosomal-lysosomal system and exhibits partial colocalization with CD63 [30]. Our previous study demonstrated that RNF149 is located in lysosomes, and in the current study, it was observed to interact with CD63 [14]. Due to their shared domain structure and similar cellular localization, it has been speculated that members of the PA-TM-RING family may have redundant or partially overlapping functions [12]. Based on the finding from Bocock et al. and our own research, we propose that RNF149 [14] and RNF13 [30] may indeed have redundant functions, requiring further investigation for confirmation.

RING domains play distinct roles in transcriptional repression, translational repression, and E3 activity [31]. E3 ubiquitin ligase activity has been identified in members of the PA-TM-RING family, including RNF128 [32], RNF13 [9], and RNF149 [13]. Understanding the substrates of these enzymes is crucial for further elucidating their functions. However, there is no specific recognition motif for the PA-TM-RING family E3 ligase [30]. Membrane proteins of the tetraspanin family, such as CD9 [14], CD151 [16], and CD81 [17], serve as substrates of PA-TM-RING family E3 ligases. In this study, we screened all tetraspanin family molecules and identified CD63 as a potential substrate of RNF149 [14], in addition to CD9. We observed that RNF149 can degrade CD63 through multiple polyubiquitination. Furthermore, the RING domain of RNF149 plays a crucial role in the ubiquitination of CD63 by covalently modifying CD63 with lysine at the 29-position of the ubiquitin monomer. RNF128 has been shown to interact with the large extracellular loops of CD151 [16] and CD81 [17]. Similarly, we hypothesize that RNF149 may interact with CD63 in the same way.

Tetraspanins are a group of membrane-organizing proteins that play essential roles in cell motility, adhesion, proliferation, invasion, and differentiation [33]. The specific mechanism involving RNF149 and CD63 remains unclear. Li and colleagues showed that CD9 and CD63 significantly increased in macrophages after stimulated with LPS [19]. Based on this observation, we hypothesize that RNF149 may be associated with inflammation, and our hypothesis was supported by bioinformatics analysis using transcriptome array data. Further examination of the IP results revealed that both RNF149 and CD63 can interact with TLR4. Kraft et al. showed that CD63-deficient bone-marrow-derived mast cells exhibit decreased TNF-α secretion [34]. TNF-α and CD63 levels increased in supernatants from LPS-treated platelets, suggesting that CD63 plays a beneficial role in the inflammatory response [35]. Consistent with Kraft et al.’s finding, the TNF-α level in the CD63 knockdown group was lower than that in the negative control group in RAW264.7 cells after LPS stimulation. Conversely, the TNF-α level increased after RNF149 knockdown, supporting our discovery that RNF149 can degrade CD63 through multiple polyubiquitination. In summary, our results indicate that RNF149 and CD63 are involved in TLR4-dependent platelet reactions. Guo et al. have recently made an important discovery regarding RNF149, revealing its role in promoting the progression of HCC through its E3 ubiquitin ligase activity [36]. Their research demonstrated that RNF149 facilitates the UPS-dependent degradation of DNAJC25 [36]. Additionally, they highlighted the association between elevated RNF149 expression and an immunosuppressive tumor microenvironment [36]. While our research focused on different substrates, the shared mechanism implies that RNF149 could participate in diverse immune responses, playing a crucial role in immunology. We then investigated the potential mechanism of CD63 in LPS/TLR4 signaling. CD63 is known for its transporter-related physiological activities, suggesting that it may play a role in regulating the distribution of TLR4 on the cell surface [22]. However, our study in RAW264.7 cells indicated that CD63 did not impact TLR4 distribution. In light of this, we propose that CD63 could enhance LPS/TLR4 signal transduction by facilitating the recruitment of downstream molecules of the TLR4 pathway [23]. The MyD88-independent pathway is known to involve TLR4 endocytosis [27], but since we observed no change in TLR4 distribution, we ruled out the involvement of the MyD88-independent pathway. IP analysis revealed an interaction between CD63 and MyD88. Furthermore, bioinformatics analysis using transcriptome data showed an increase in NF-κB and JUN, and a decreased in IRF3 levels following LPS stimulation, supporting the idea of a MyD88-dependent pathway. Notably, although there were only slight differences in specific expression values, MyD88 levels actually decreased somewhat after LPS stimulation, rather than increasing. This result suggests that excessive immune stress might be controlled by inhibiting MyD88, or that modifications to MyD88 after LPS stimulation could influence the corresponding immune response, rather than simply increasing its quantity. Further experimental investigations are needed to fully elucidate the specific mechanism of action. Zhu et al. discovered that esophageal squamous cell carcinoma cells become resistant to cisplatin when RNF149 is overexpressed. Conversely, inhibiting RNF149 reversed this resistance both in vitro and in vivo [37]. Their research showed that RNF149 interacts with PH domain and leucine-rich repeat protein phosphatase 2 (PHLPP2), leading to E3 ligase-dependent degradation of PHLPP2. This process significantly activates the PI3K/AKT signaling pathway in ESCC [37]. Our results, along with those of Zhu et al., suggest that RNF149 may exert different physiological effects through different pathways.

## Compliance with ethical standards

The authors declare that they have no competing interests.

## Data availability statement

The datasets used and/or analyzed during the current study are available from the corresponding author upon reasonable request.

## CRediT authorship contribution statement

**Xiu-An Yang:** Writing – original draft, Supervision, Methodology, Funding acquisition, Formal analysis, Conceptualization. **Yingying Wang:** Investigation. **Mingyu Gong:** Investigation. **Zicheng Zhao:** Investigation. **Fengchun Lv:** Investigation. **Xiaoyu Zhang:** Writing – review & editing. **Yan Li:** Writing – review & editing, Writing – original draft, Supervision, Methodology, Investigation, Formal analysis, Data curation, Conceptualization.

## Declaration of competing interest

The authors declare that they have no known competing financial interests or personal relationships that could have appeared to influence the work reported in this paper.

## Figures and Tables

**figs1:**