# Supplementary material for: RNF149 negatively regulates LPS/TLR4 signal transduction by ubiquitination-mediated CD63 degradation
Source: Heliyon. 2024 Jul 11;10(14):e34350. doi: 10.1016/j.heliyon.2024.e34350 (PMC11298846; doi:10.1016/j.heliyon.2024.e34350)
Supplement: Multimedia component 1 [file mmc1.docx]

**Fig 2**


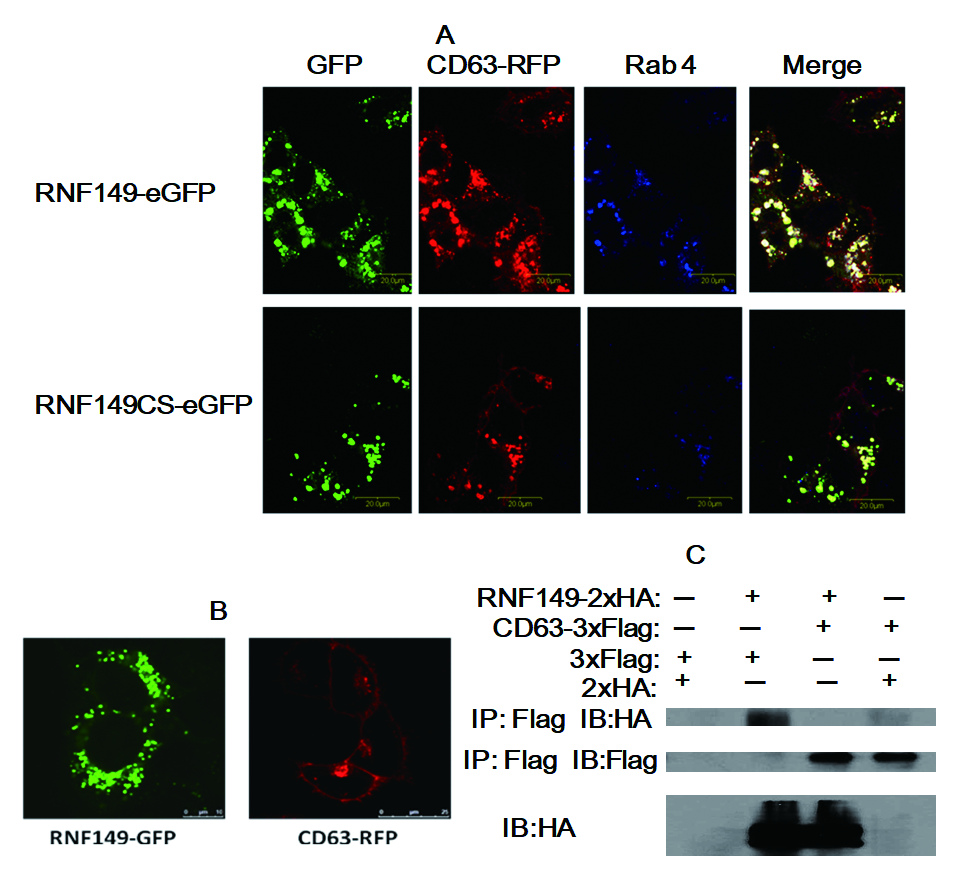

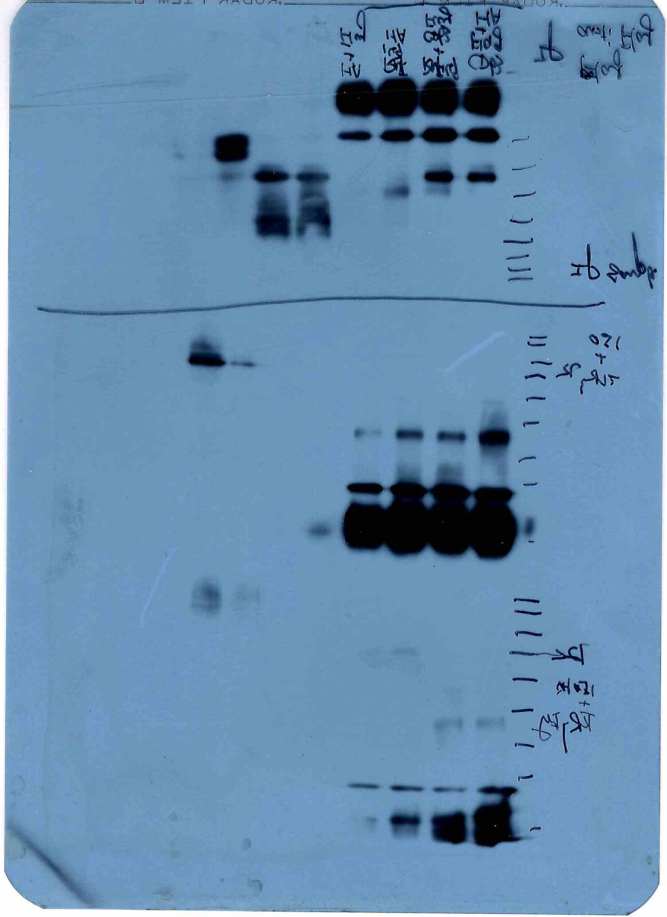


**Fig2C-2**


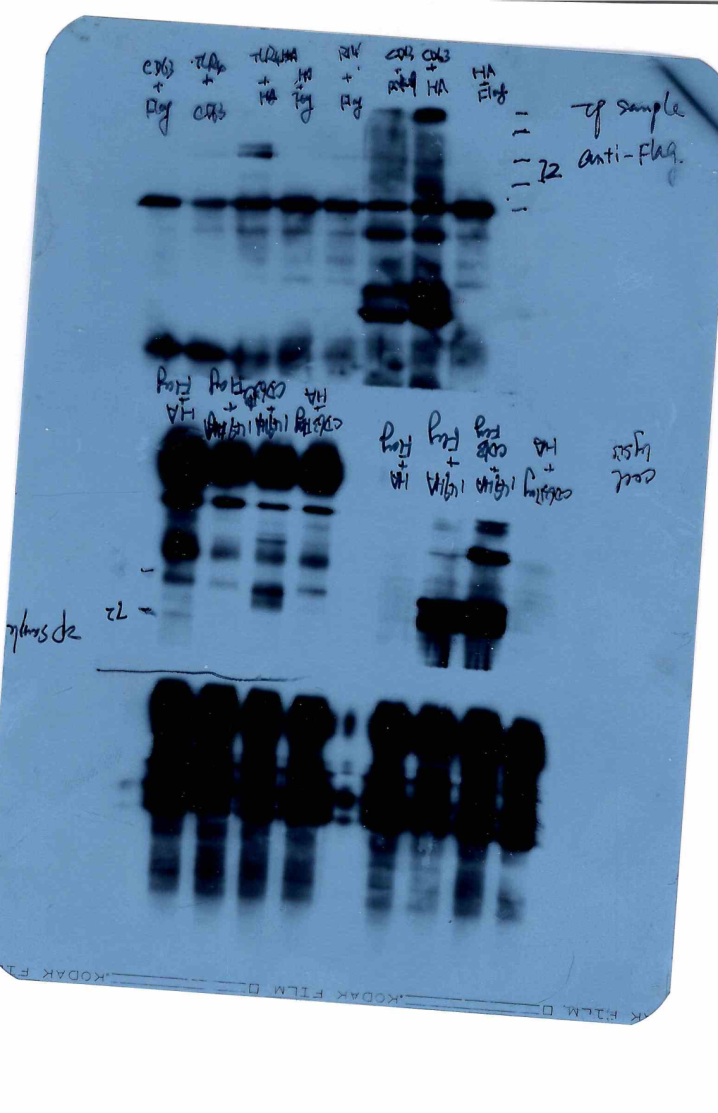


**Fig2C-1**

**Fig3A**


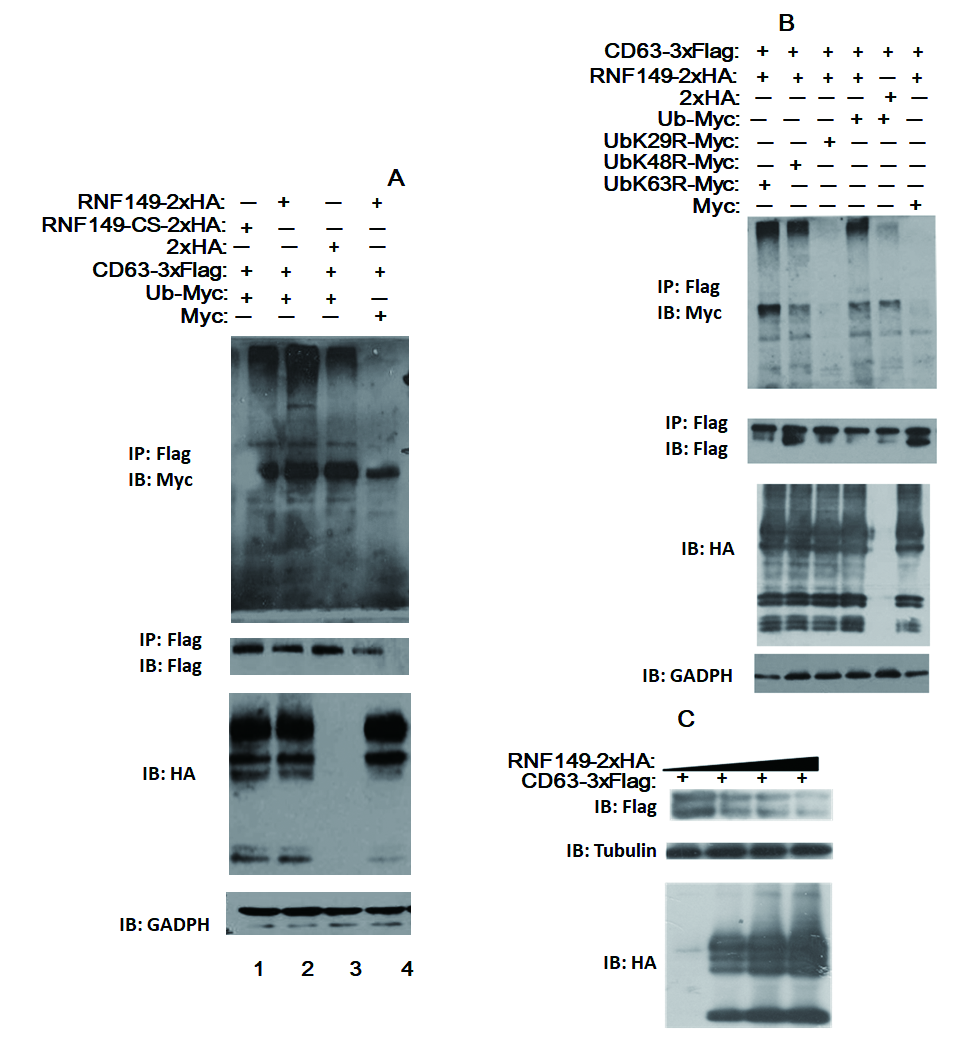

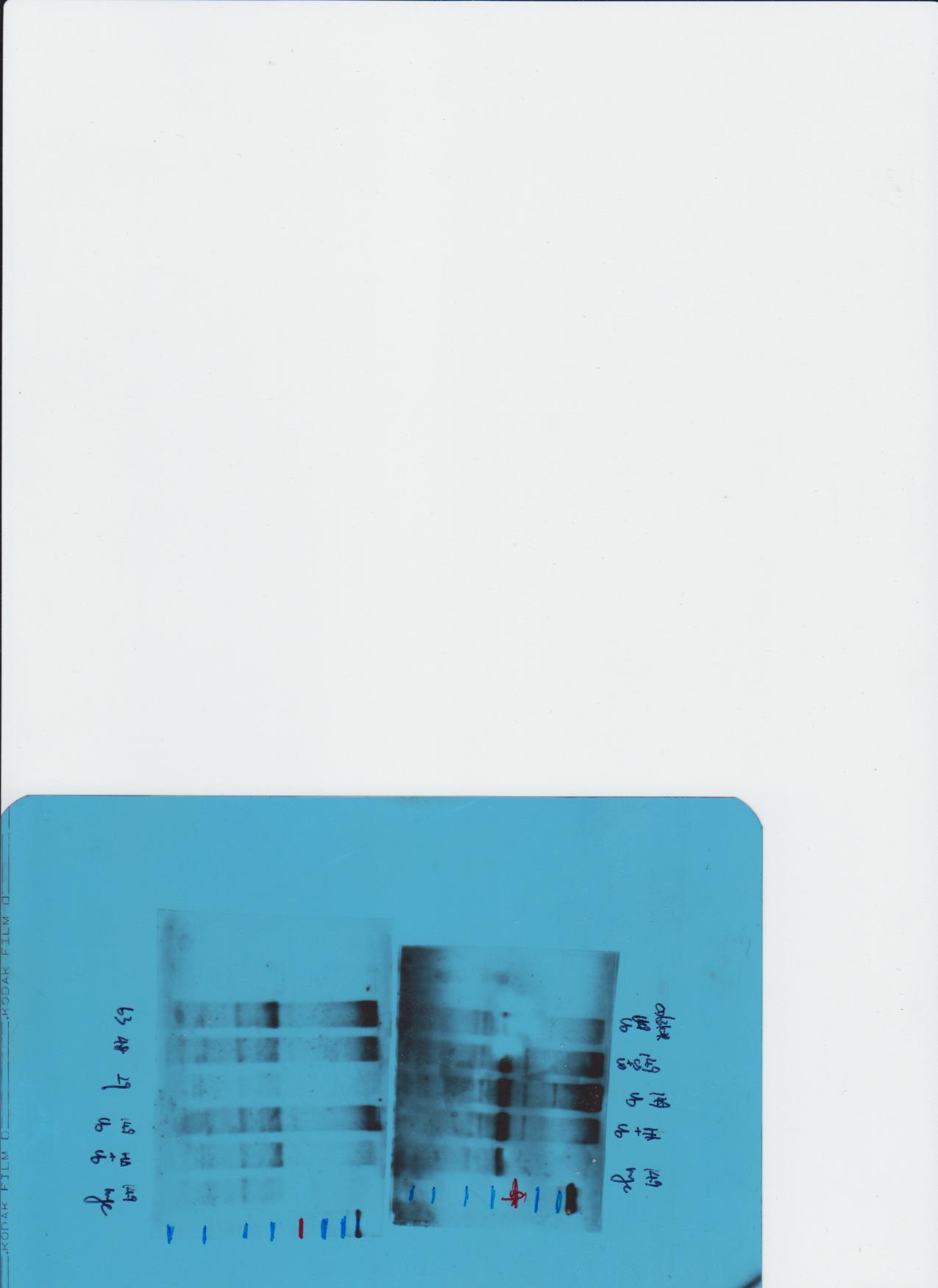


**Fig3A-1**


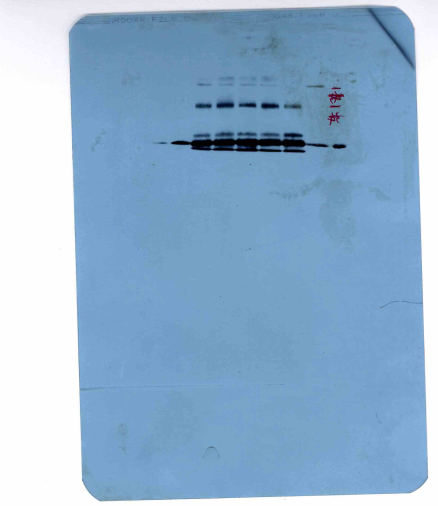

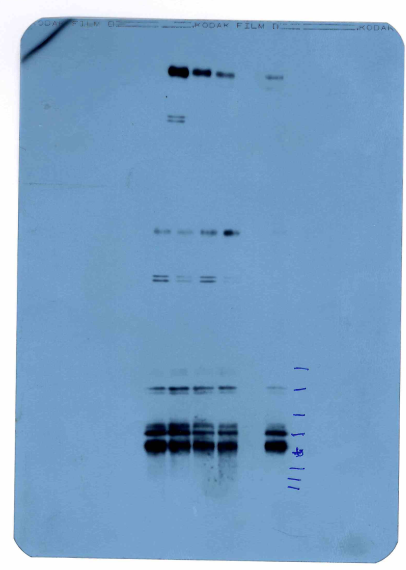

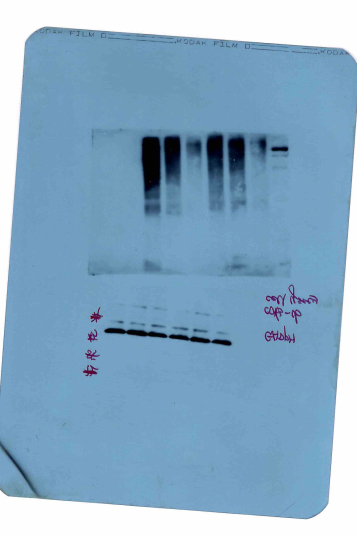


**Fig3A-4**

**Fig3A-3**

**Fig3A-2**

**Fig3B**

**
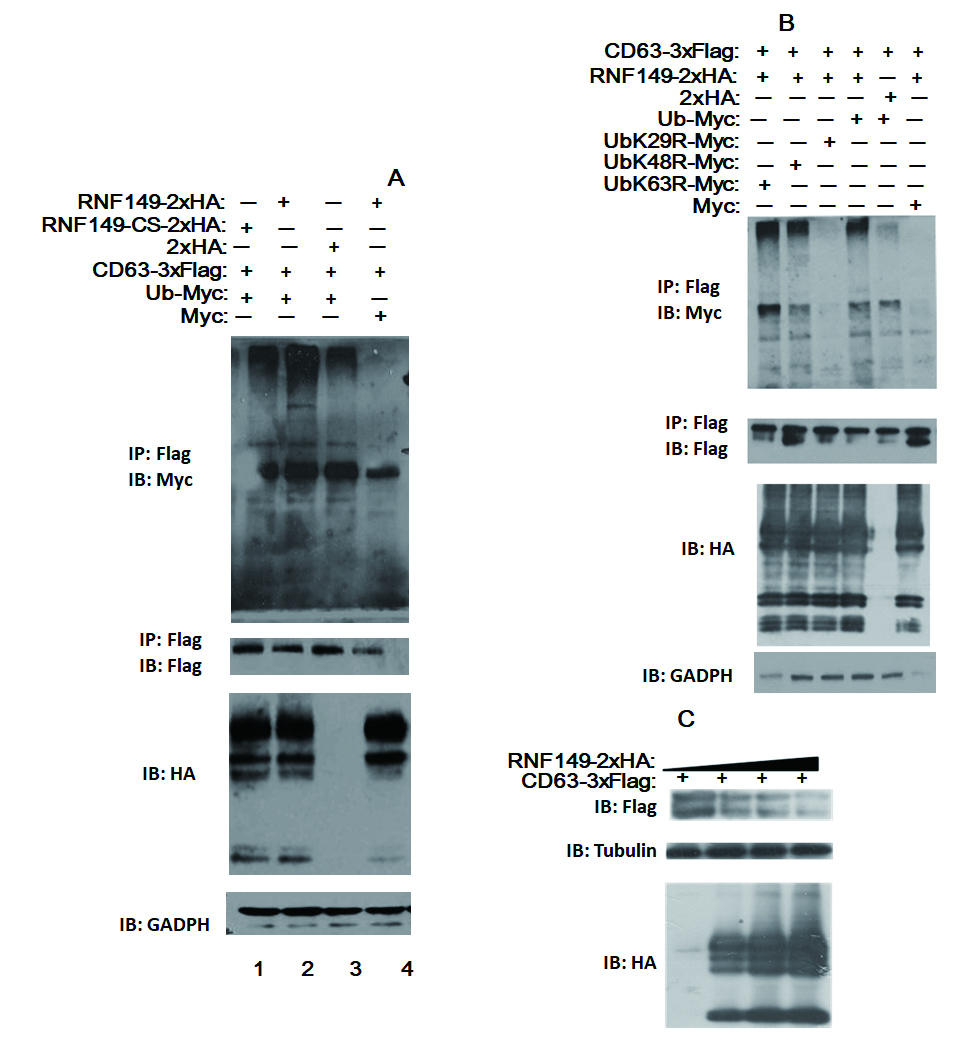

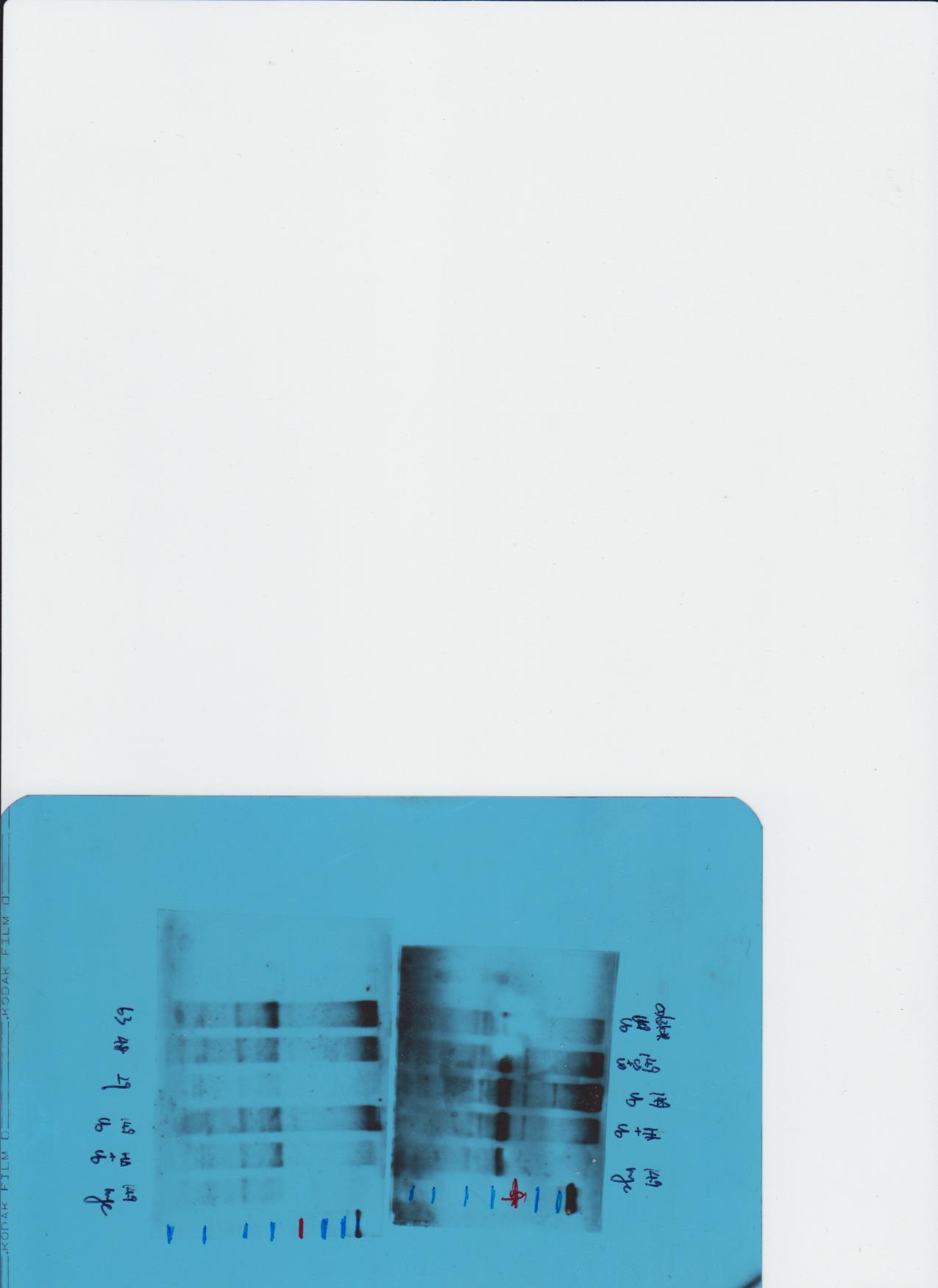
**

**Fig3B-2**

**Fig3B-1**

**
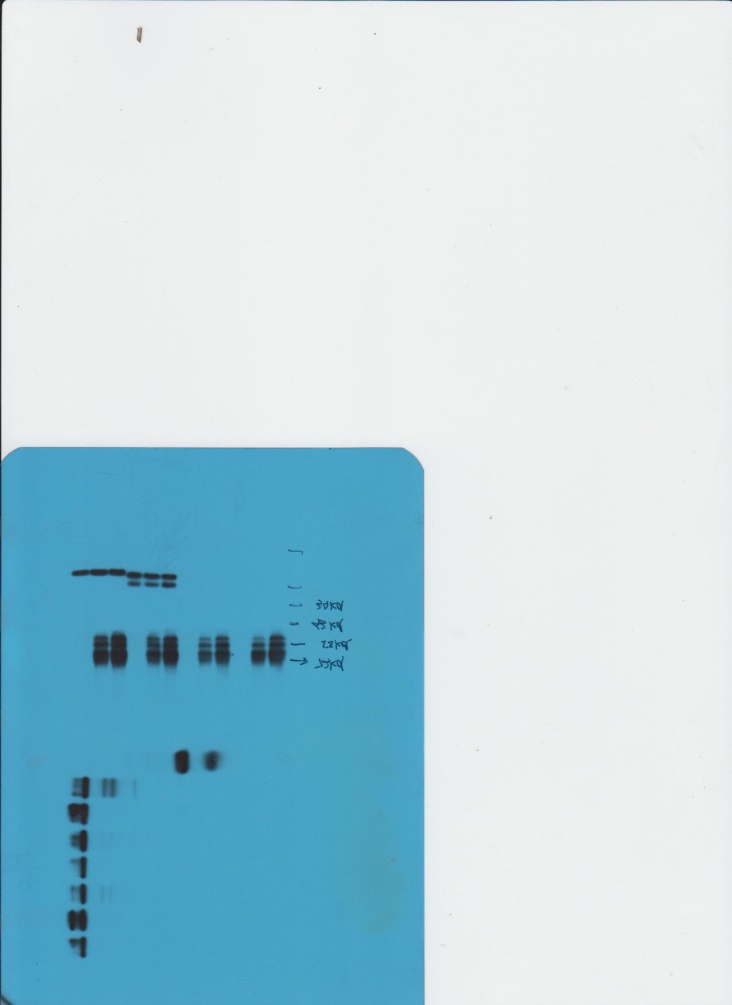

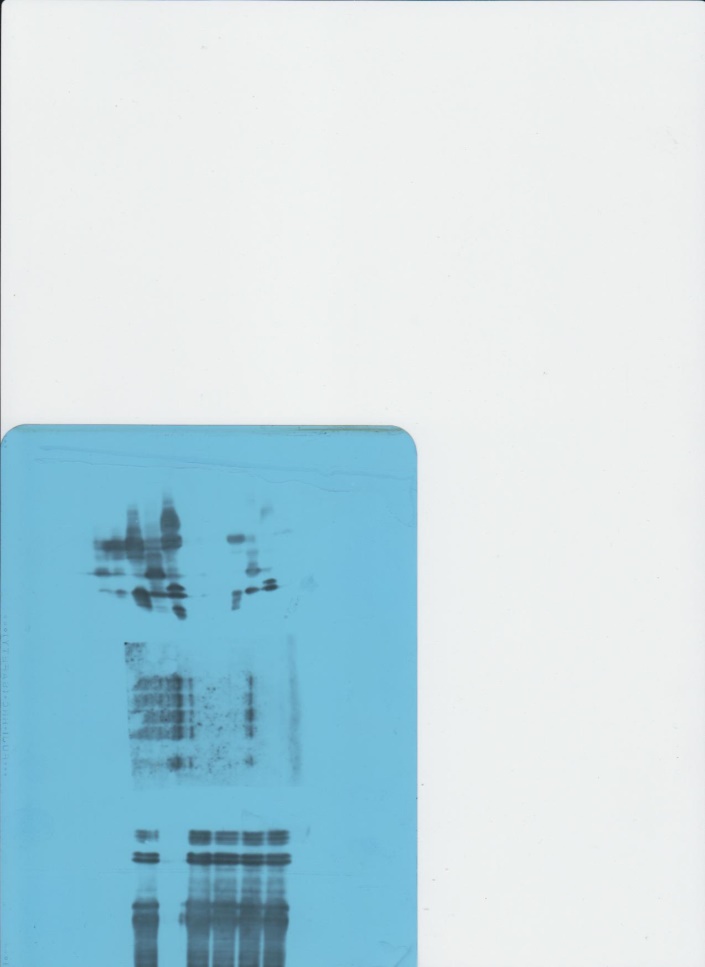

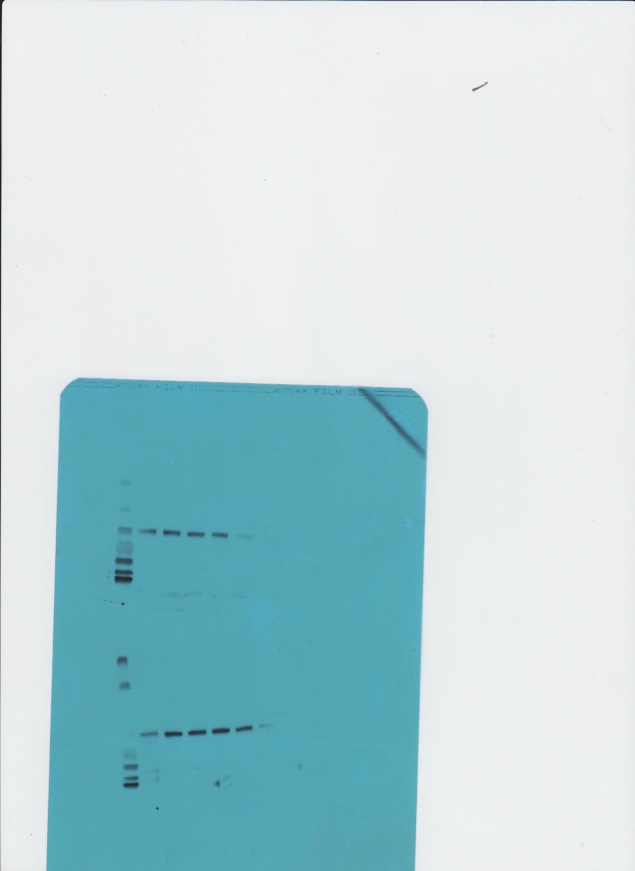
**

**Fig3B-4**

**Fig3B-3**

**Fig3C**

**
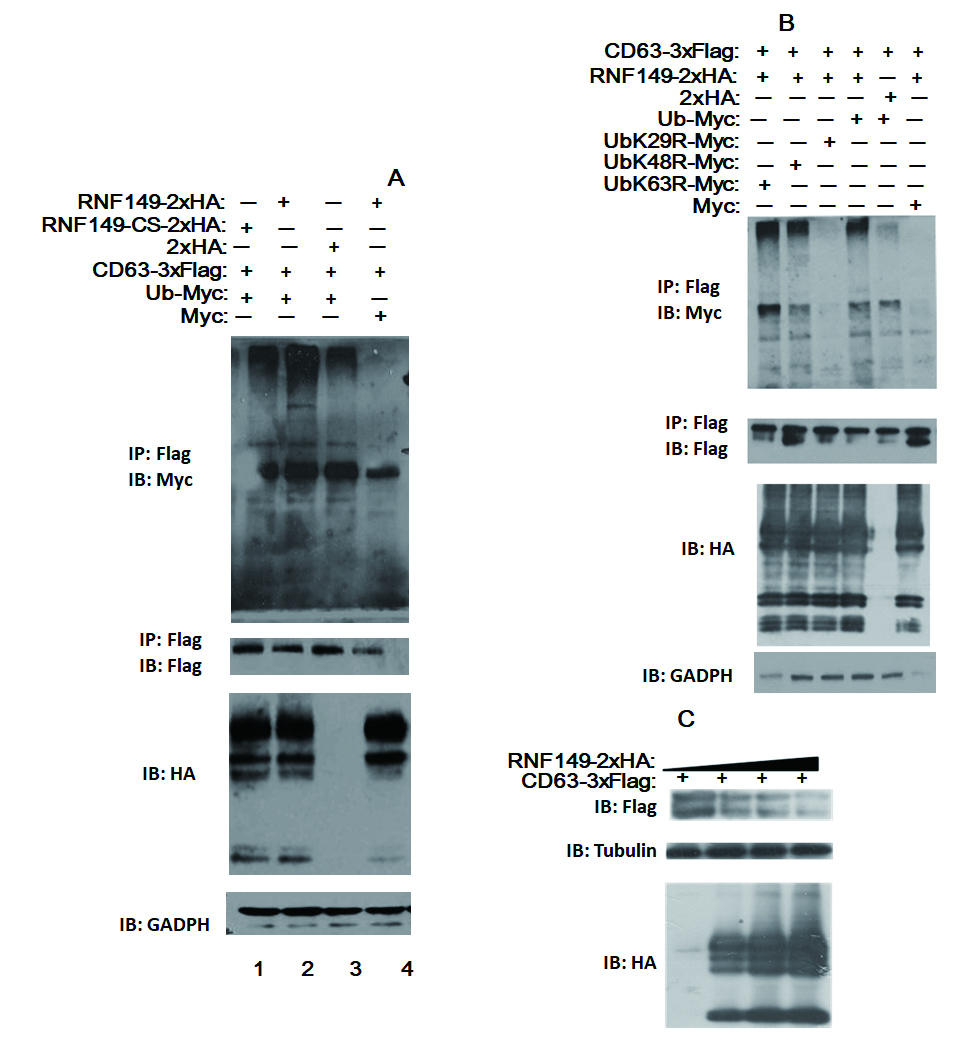

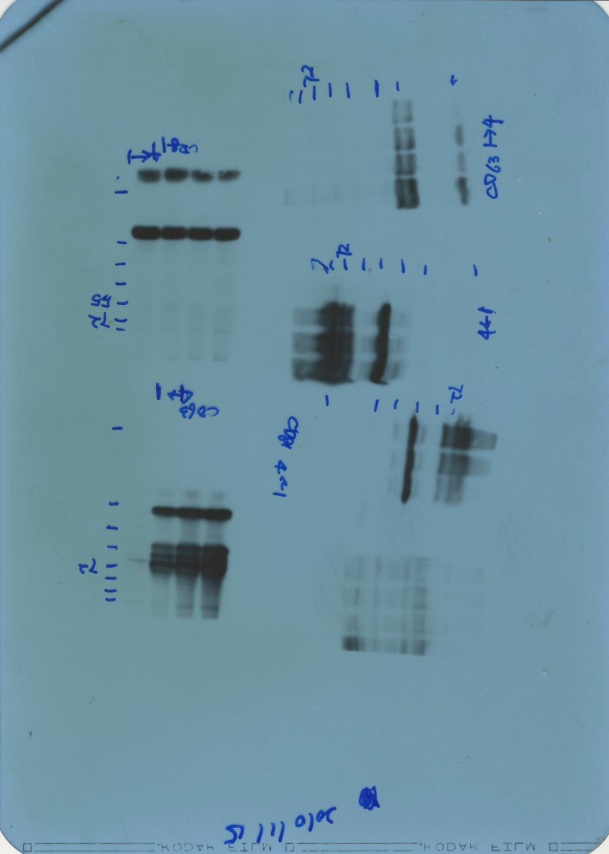
**

**Fig3C-1**

**
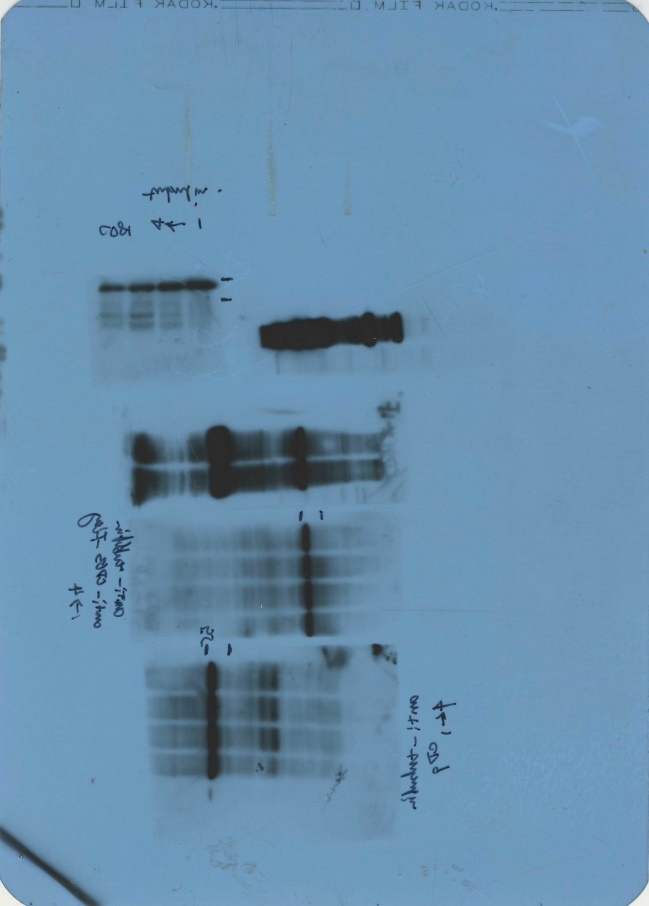
**

**Fig3C-2**

**Fig4C**

**
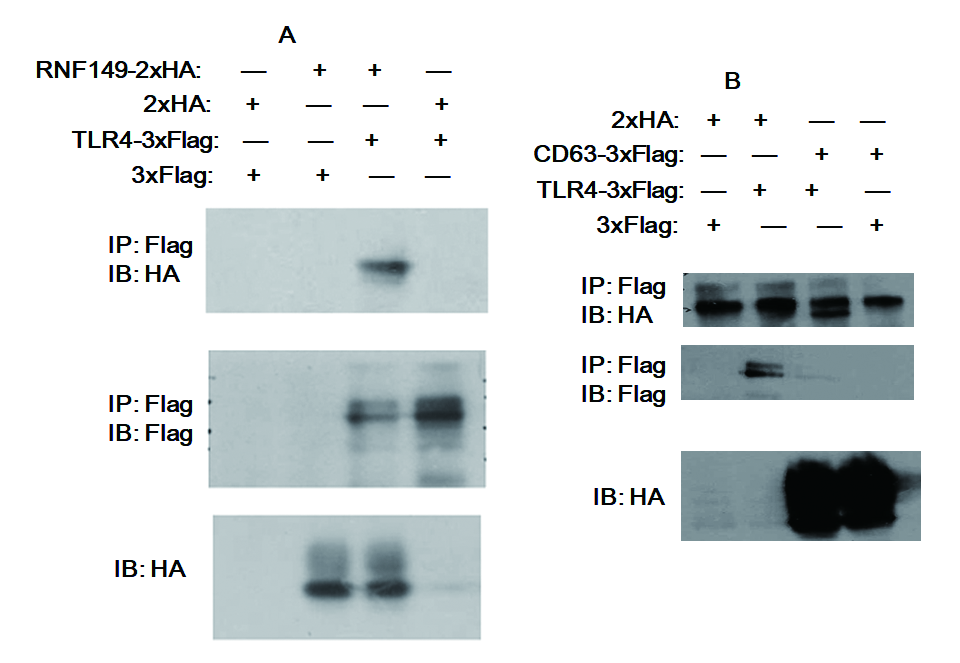

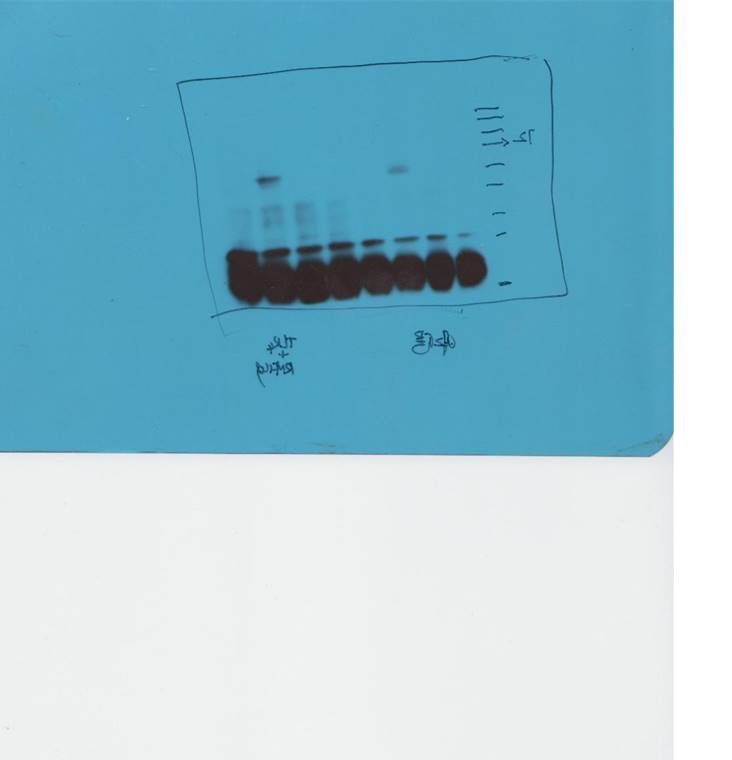
**

**Fig4C-1**

**
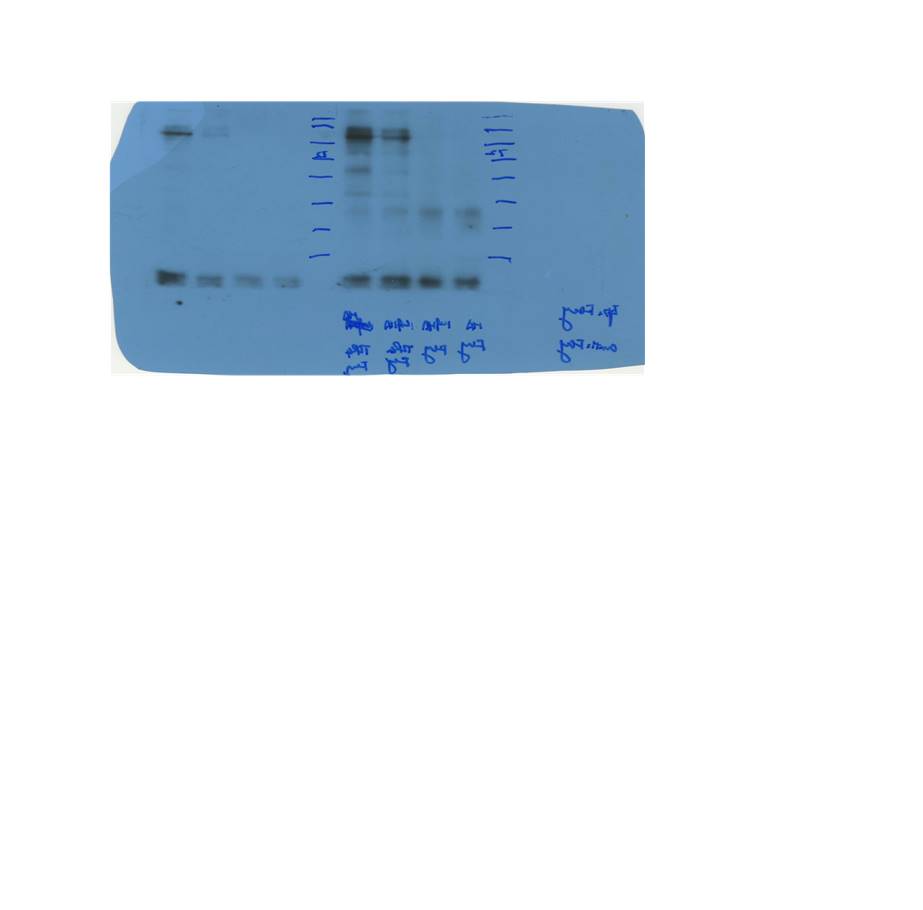

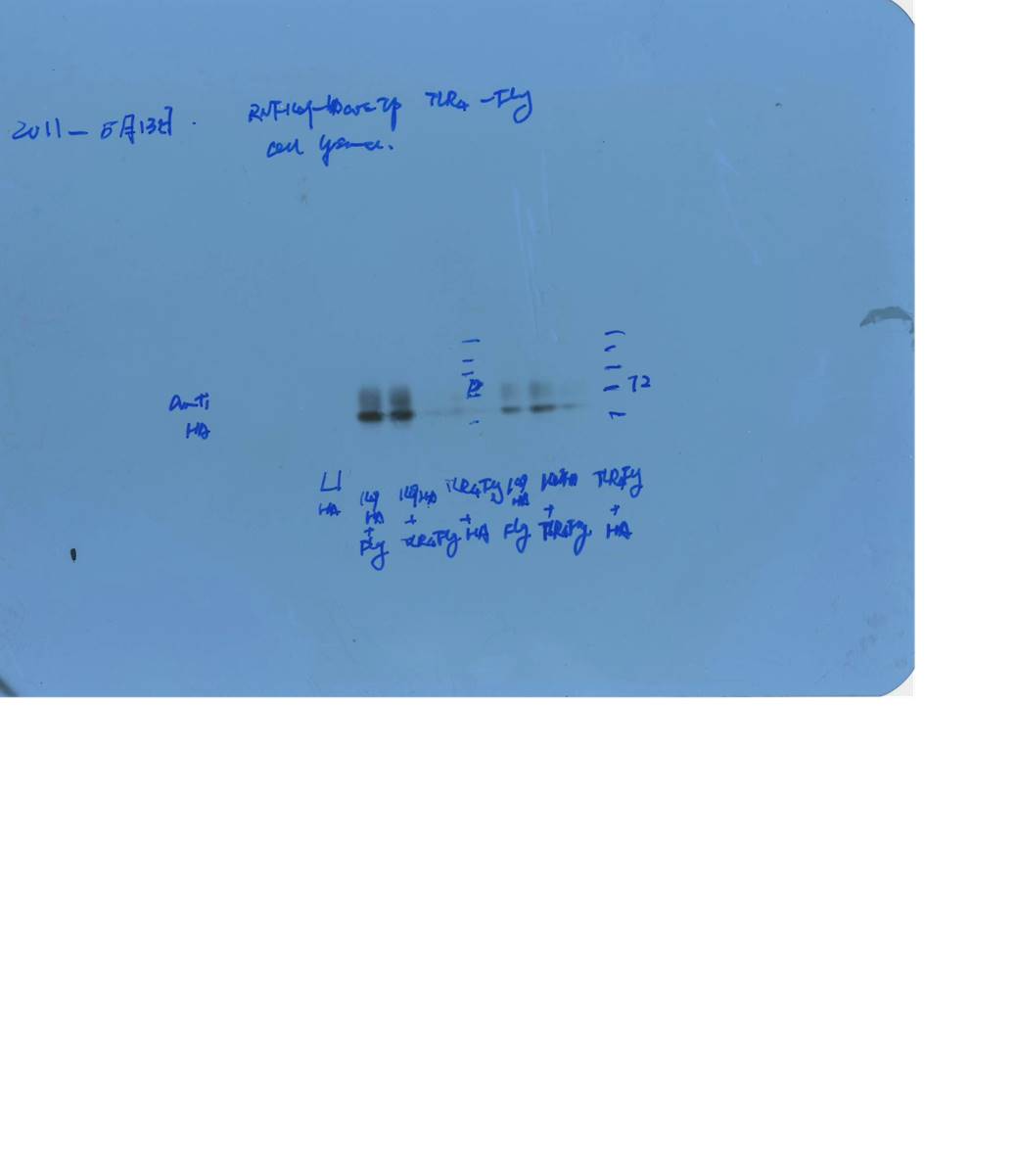
**

**Fig4C-3**

**Fig4C-2**

**Fig4D**

**
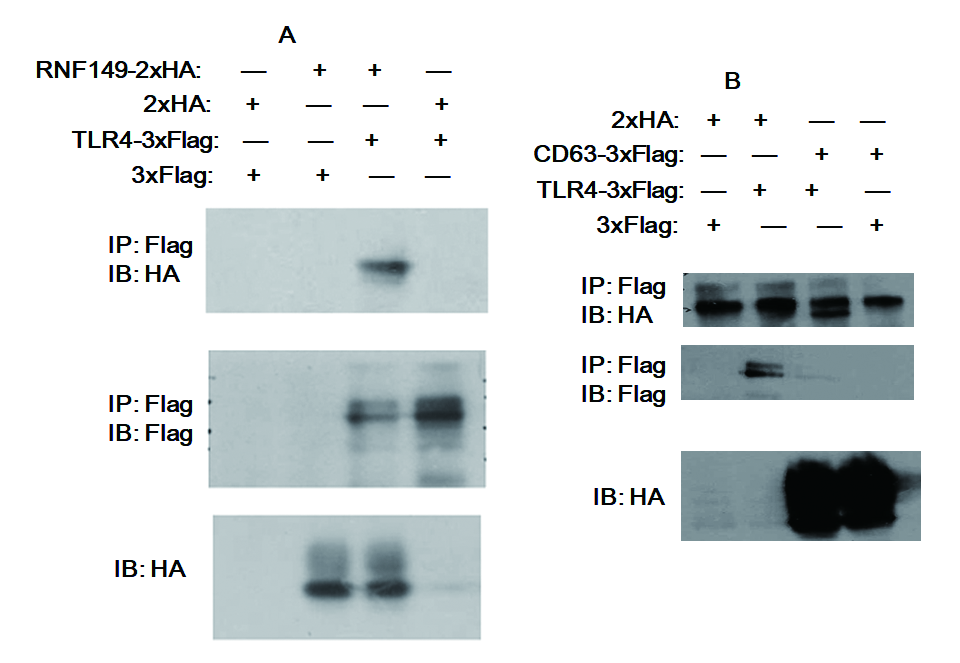

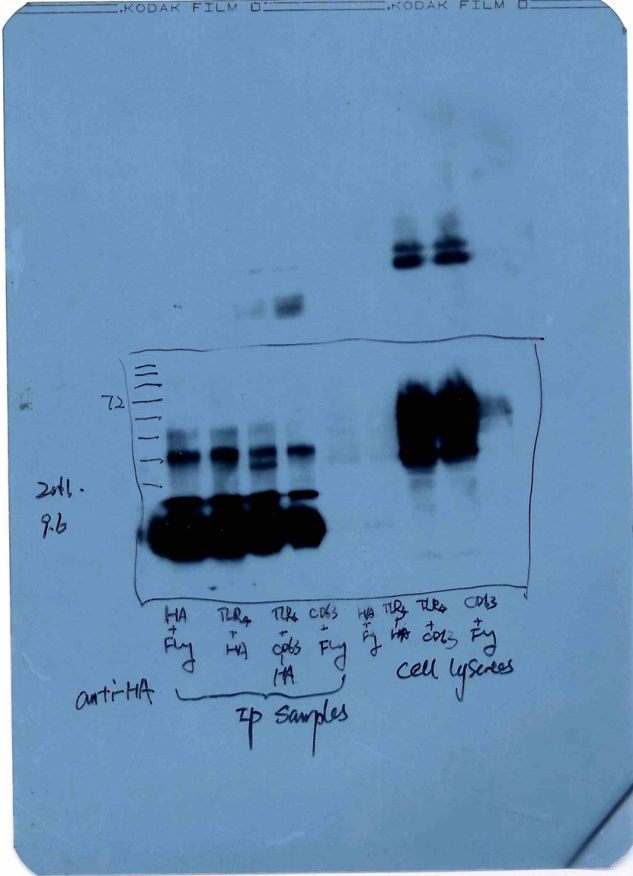

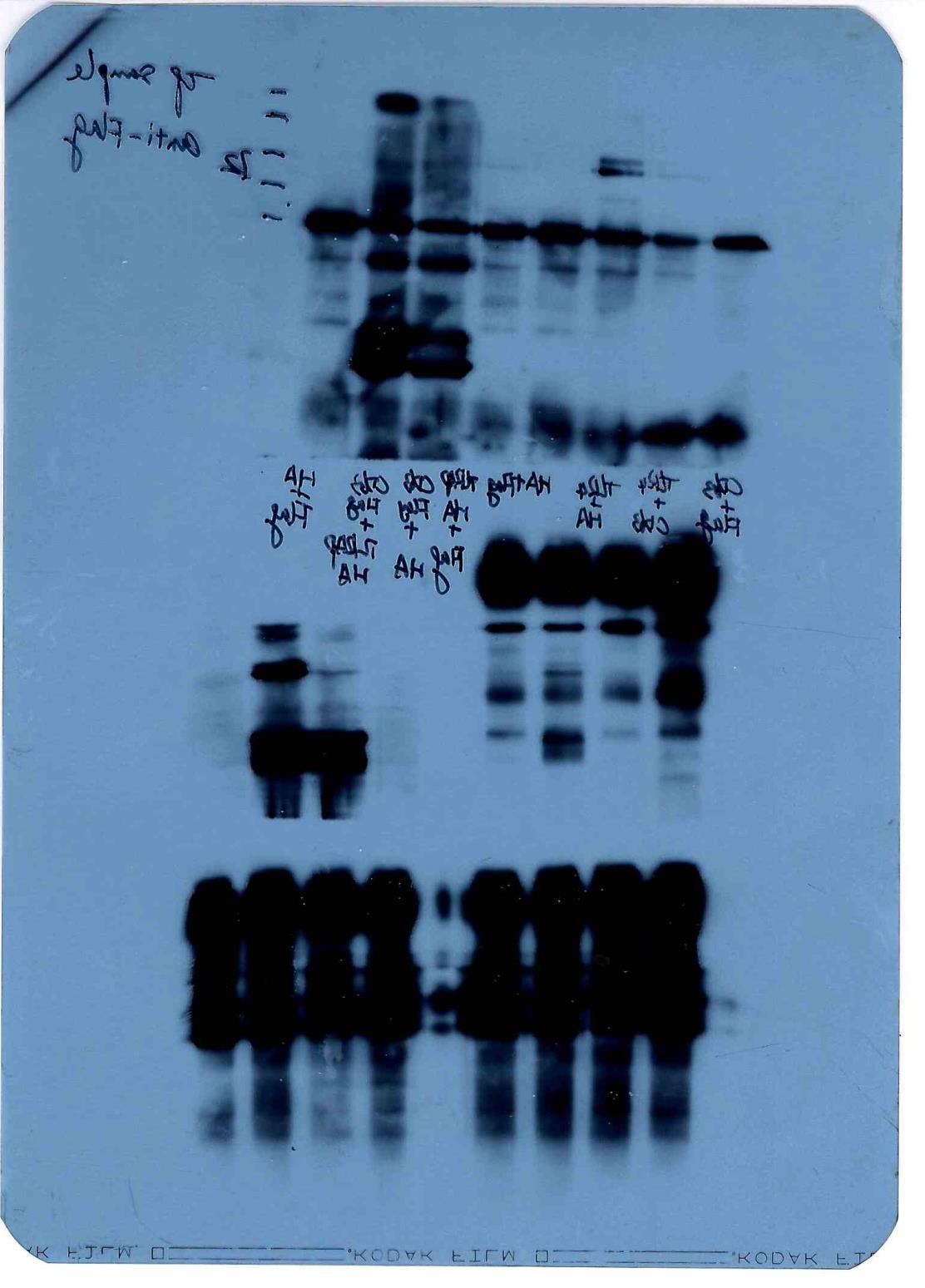
**

**Fig4D-2**

**Fig4D-1**

**Fig6B**


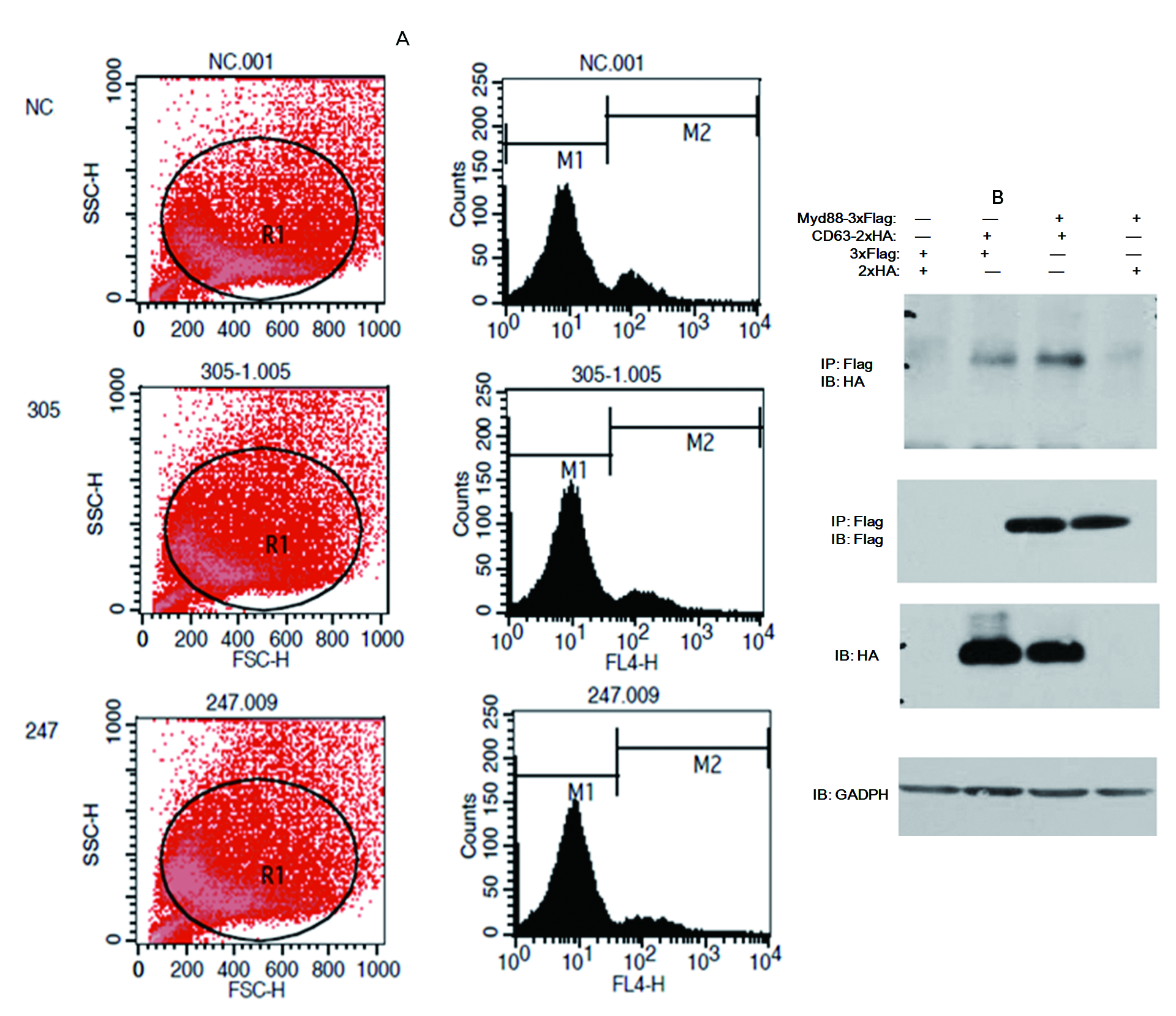


**Fig6B-1**


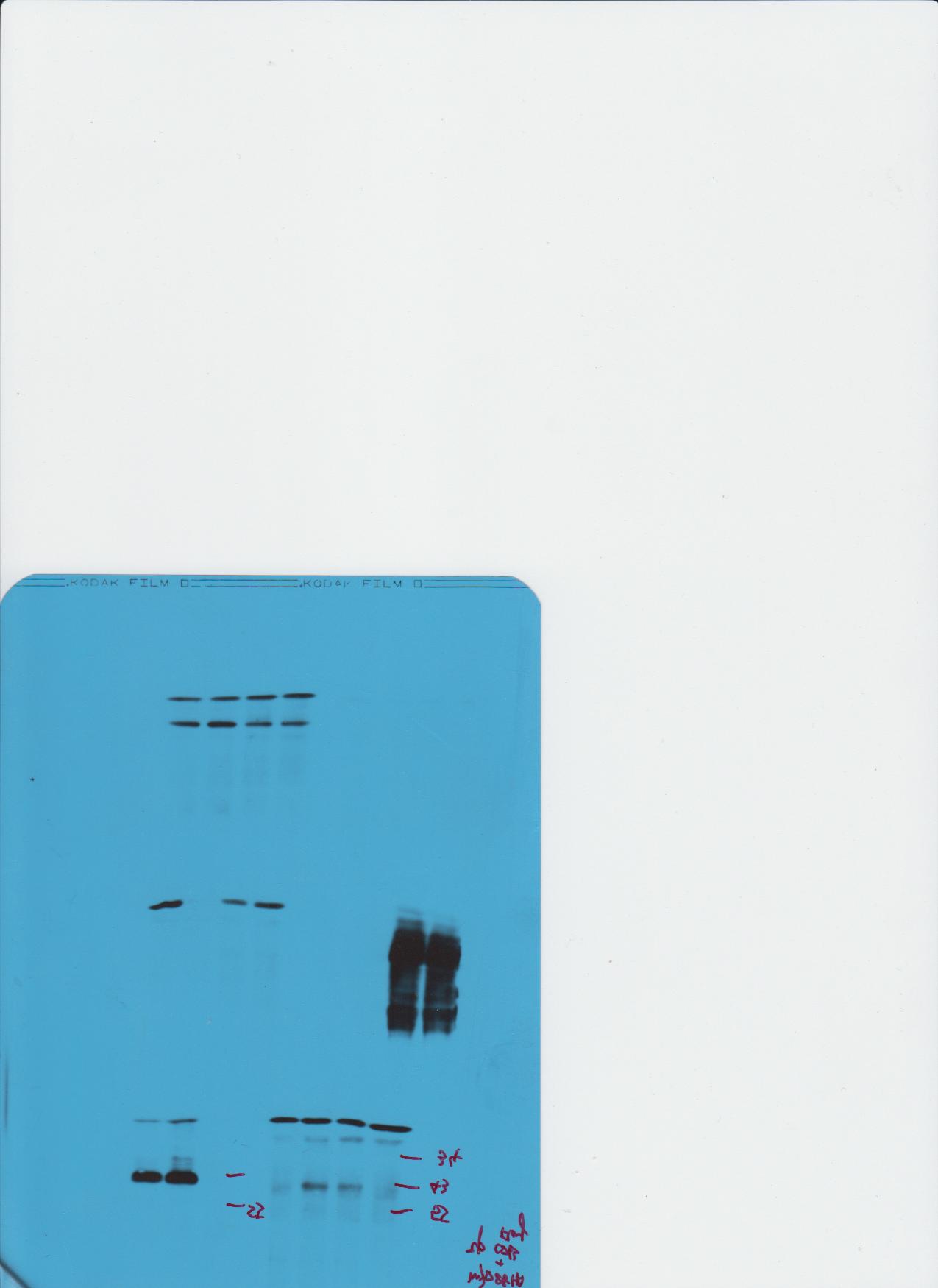

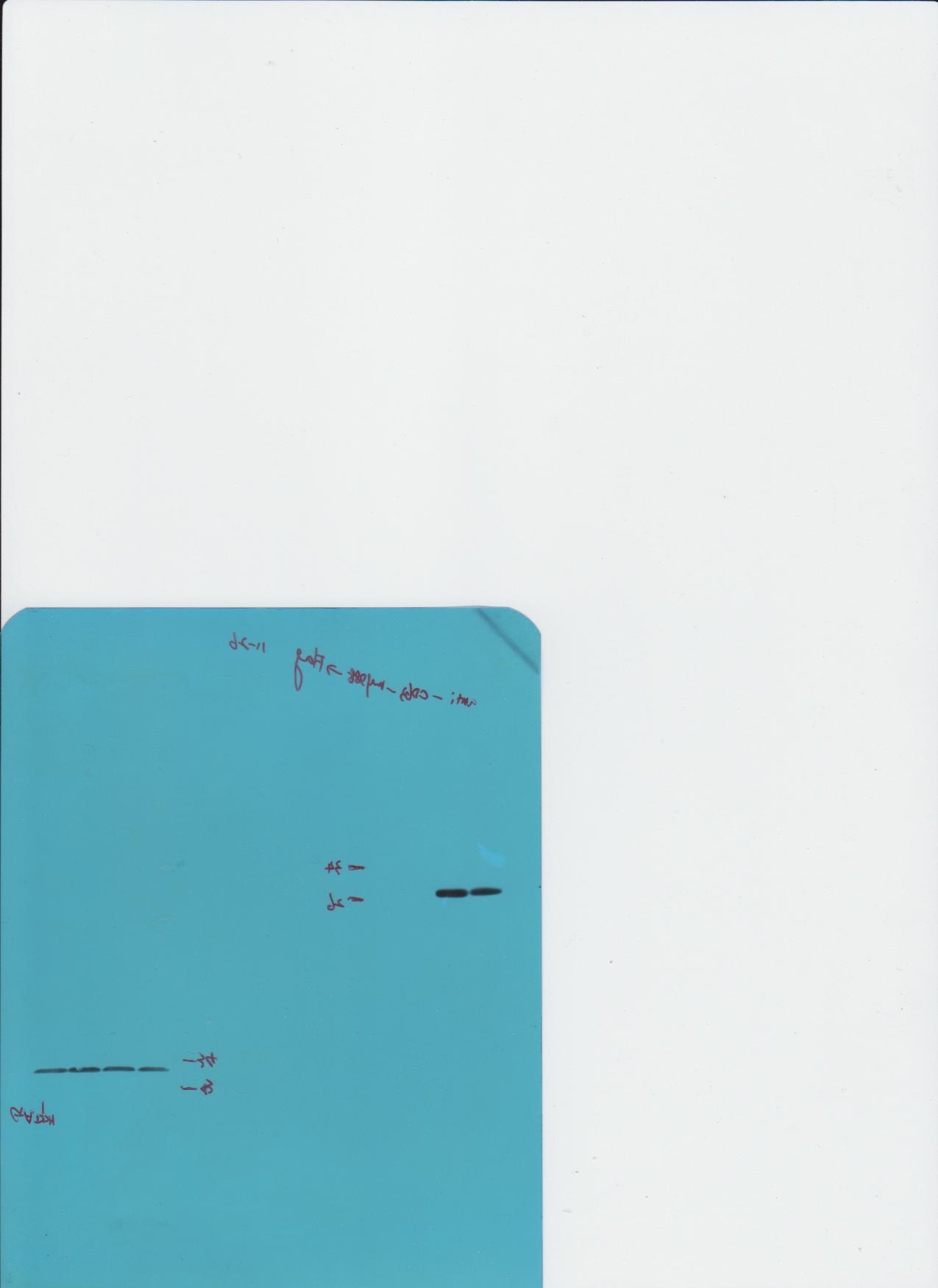


**Fig6B-2**
